# Genome-wide RNA sequencing of ocular fibroblasts from glaucomatous and normal eyes: Implications for glaucoma management

**DOI:** 10.1371/journal.pone.0307227

**Published:** 2024-07-11

**Authors:** Anton W. Roodnat, Breedge Callaghan, Chelsey Doyle, Neeru A. Vallabh, Sarah D. Atkinson, Colin E. Willoughby

**Affiliations:** 1 Centre for Genomic Medicine, Biomedical Sciences Research Institute, Ulster University, Coleraine, Northern Ireland, United Kingdom; 2 Department of Eye and Vision Science, Insitute of Life Course and Medical Sciences, University of Liverpool, Liverpool, United Kingdom; 3 St. Paul’s Eye Unit, Liverpool University Hospital NHS Foundation Trust, Liverpool, United Kingdom; Oregon Health and Science University, UNITED STATES OF AMERICA

## Abstract

Primary open angle glaucoma is a leading cause of visual impairment and blindness which is commonly treated with drugs or laser but may require surgery. Tenon’s ocular fibroblasts are involved in wound-healing after glaucoma filtration surgery and may compromise a favourable outcome of glaucoma surgery by contributing to fibrosis. To investigate changes in gene expression and key pathways contributing to the glaucomatous state we performed genome-wide RNA sequencing. Human Tenon’s ocular fibroblasts were cultured from normal and glaucomatous human donors undergoing eye surgery (n = 12). mRNA was extracted and RNA-Seq performed on the Illumina platform. Differentially expressed genes were identified using a bioinformatics pipeline consisting of FastQC, STAR, FeatureCounts and edgeR. Changes in biological functions and pathways were determined using Enrichr and clustered using Cytoscape. A total of 5817 genes were differentially expressed between Tenon’s ocular fibroblasts from normal versus glaucomatous eyes. Enrichment analysis showed 787 significantly different biological functions and pathways which were clustered into 176 clusters. Tenon’s ocular fibroblasts from glaucomatous eyes showed signs of fibrosis with fibroblast to myofibroblast transdifferentiation and associated changes in mitochondrial fission, remodeling of the extracellular matrix, proliferation, unfolded protein response, inflammation and apoptosis which may relate to the pathogenesis of glaucoma or the detrimental effects of topical glaucoma therapies. Altered gene expression in glaucomatous Tenon’s ocular fibroblasts may contribute to an unfavourable outcome of glaucoma filtration surgery. This work presents a genome-wide transcriptome of glaucomatous versus normal Tenon’s ocular fibroblasts which may identify genes or pathways of therapeutic value to improve surgical outcomes.

## Introduction

Glaucoma is a progressive irreversible optic neuropathy characterised by loss of retinal ganglion cells ultimately leading to blindness if untreated [1]. The commonest form of glaucoma is primary open angle glaucoma (POAG) which had an estimated prevalence of roughly 55 million in 2020 [2, 3]. Risk factors for POAG include age, sex, genetic background and increased intraocular pressure (IOP); which is an important modifiable risk factor [4, 5]. POAG shows signs of a fibrotic condition [6, 7] and is associated with increased levels of profibrotic cytokine TGF-β2 in the aqueous humour [8].

Initial treatment of POAG commonly consists of SLT laser treatment or drugs intended to lower the IOP such as prostaglandins, β-adrenergic inhibitors and carbonic anhydrase inhibitors [9]. These drugs are usually applied topically in the form of eyedrops and frequently contain preservatives such as benzalkonium chloride (BAC). Unfortunately, BAC is toxic to the eye and has been associated with fibrosis, cell death, mitochondrial dysfunction, the generation of reactive oxygen species and DNA damage [9, 10]. During treatment of POAG surgical intervention in the form of glaucoma filtration surgery may be required. The failure rate of this procedure can be as high as 35% when evaluated after 5 years [11] and success depends on modulation of the wound healing response and prevention of fibrosis using anti-proliferative and anti-fibrotic drugs, such as 5-fluorouracil and mitomycin-C [12].

One cell type that is of particular importance in the postoperative wound-healing response is the Tenon’s ocular fibroblast (TF; also called Tenon’s capsule fibroblast) [13] because it is a main source of pro-fibrotic myofibroblasts involved in ECM deposition and wound contraction which may lead to scarring and subsequent bleb failure [12, 14, 15].

Previously it was shown that TFs from glaucomatous patients show signs of fibrosis when compared to non-glaucomatous patients by considering differential expression of a limited predefined panel of 10 genes [16]. The main purpose of this study was to perform a hypothesis-independent genome-wide RNA sequencing analysis to obtain a more complete view of the alterations in the transcriptome of glaucomatous Tenon’s ocular fibroblast (GTF) cells when compared to normal Tenon’s ocular fibroblasts (NTFs).

## Materials and methods

### Tenon’s ocular fibroblasts culturing and characterisation

Primary human Tenon’s ocular fibroblasts (TFs) were obtained from patients with POAG and non-glaucomatous patients during the period 06 Oct 2015 until 28 Feb 2017 using a previously described standardised method [17, 18]. Patient information can be found in S3 Table. Cells were maintained in low glucose Dulbecco`s Modified Eagle Media (DMEM) (Sigma, Gillingham, UK) which was supplemented with 10% fetal calf serum (Biosera, Heathfield, UK) and 2mM L-glutamine (Sigma, Gillingham, UK), Penicillin-Streptomycin (Sigma, Gillingham, UK), and 2.5 μg/mL Fungizone (amphotericin B, Sigma, Gillingham, UK). The cells were incubated at 37.0 °C, 95 % humidity and 5 % CO_2_. Cells were sub-cultured after reaching roughly 70 % confluency; cultures were used up to passage four. Tenon’s ocular fibroblast characterisation was carried out by fluorescent staining using a mouse monoclonal antibody (Thermofisher Scientific, USA) against vimentin which is a fibroblast biomarker. Bright focus microscopy and fluorescent vimentin and DAPI staining cell images of these Tenon’s ocular fibroblasts were shown previously in another publication by our group in [19].

### RNA extraction

Total RNA was obtained from the cultured Tenon’s ocular fibroblasts using the Qiagen RNeasy Mini Kit (Qiagen, Manchester UK) following the manufacturer’s instructions. RNA concentration was determined using the NanoDrop 2000 instrument (Thermofisher Scientific, Horsham, UK). RNA quality was assessed using the Bioanalyser 2100 (Agilent Technologies, Stockport, UK) combined with an RNA 6000 Nano Kit (Agilent, Santa Clara, CA, USA).

### RNA sequencing

Generation of sequencing libraries was achieved using the NEBNext Ultra TM RNA library Prep Kit (NEB, Ipswich, MA, USA). Purification of mRNA was performed using magnetic poly-T oligo-attached beads. RNA sequencing was done by Genewiz (Genewiz, Bishop’s Stortford, UK) using an Illumina platform thus producing a set of FASTQ files containing paired end reads.

### RNA-Seq data analysis

Quality control of the resulting paired end reads was performed using program FastQC v0.11.9 [20]. Adaptor removal and quality trimming of reads was done using Cutadapt 3.0 [21] in a linux environment thus producing high-quality trimmed reads with a Phred quality score larger than 25 which corresponds to <0.3% base calling error rate. The high-quality adapter-trimmed reads were aligned to the primary assembly GRCh38 of the human genome sequence using the STAR aligner 2.7.6a [22]. The source of the primary assembly GRCh38 was the GENCODE comprehensive gene annotation for the primary assembly [23]. To quantify reads per feature (i.e. gene) the program FeatureCounts 2.0.1 [24] was utilised. This resulted in a table containing counts per sample and feature/gene. To reduce potential mapping false positives, genes with an average count-value lower than 100 were removed. Next, the filtered counts table was used as input to determine differential gene expression which was done using the program edgeR version 3.32.1 [25] in R studio. This procedure resulted in a gene list containing false discovery rate (FDR), p-value and counts per million reads mapped (CPM). Genes with an absolute |log_2_FC| > 0.26 (which corresponds to at least 20%-fold change) and FDR < 0.05 were considered to be differentially expressed genes (DEGs). These DEGs were used for subsequent analysis.

### Functional enrichment analysis

Using the resulting list of DEGs, a functional analysis was done using enrichment tool Enrichr [26]. Enrichr utilises a modified Fisher exact test to determine over-representation of an input gene list in a.o. gene ontologies and pathway databases. Using Enrichr, functional and pathway enrichment was determined for pathway databases WikiPathways, KEGG, Reactome, MSigDB Hallmark genesets and furthermore for Gene Ontology (GO) biological processes and molecular functions. Pathways or GO terms were assumed to be enriched if the false discovery rate FDR < 0.01. The resulting enrichment terms tend to show a large amount of overlap and redundancy which can be partially removed using clustering. To cluster and visualise the functional enrichment results, EnrichmentMap 3.3.4 [27] was used in Cytoscape (version 3.9.1) combined with AutoAnnotate [28]. To further visualise and investigate interesting enriched KEGG pathways, pathway gene annotation was performed using Pathview 1.30.1 [29] in R. In a similar fashion, visualisation of gene expression in pathways from WikiPathways was achieved using PathVisio [30].

### Ethics statement

The study was conducted in accordance with the Declaration of Helsinki, and ethics approval was obtained from the UK National Research Ethics Service Committee London–Hampstead (IRAS ID 133402; REC reference 14/LO/1088). Written consent was obtained from all patients.

## Results

### Descriptive features of RNA-Seq data

RNA sequencing of 12 samples that were obtained from 12 different donors resulted in 24.8M 150 bp reads per sample on average. Usually the initial Phred quality score was larger than 25 (which corresponds to a <0.3% base calling error rate) but some reads required modest quality trimming which was done using the program Cutadapt thus arriving at quality scores which were guaranteed to be larger than 25. These paired trimmed high-quality reads were mapped to the genome (GRCh38) with an average mapping percentage of 90%.

### Differential gene expression

Using the primary comprehensive gene annotation, paired trimmed high-quality reads were aligned to the genome sequence of primary assembly GRCh38. Genes with an average low number of mapped counts < 100 were filtered out. This procedure resulted in a counts-table for 11,227 aligned genes which were subsequently used to determine differentially expressed genes using edgeR in Rstudio. Using this program, fold-change (log_2_FC), log_2_CPM, false discovery rate (FDR) and p-value per gene were determined.

**Fig 1:**
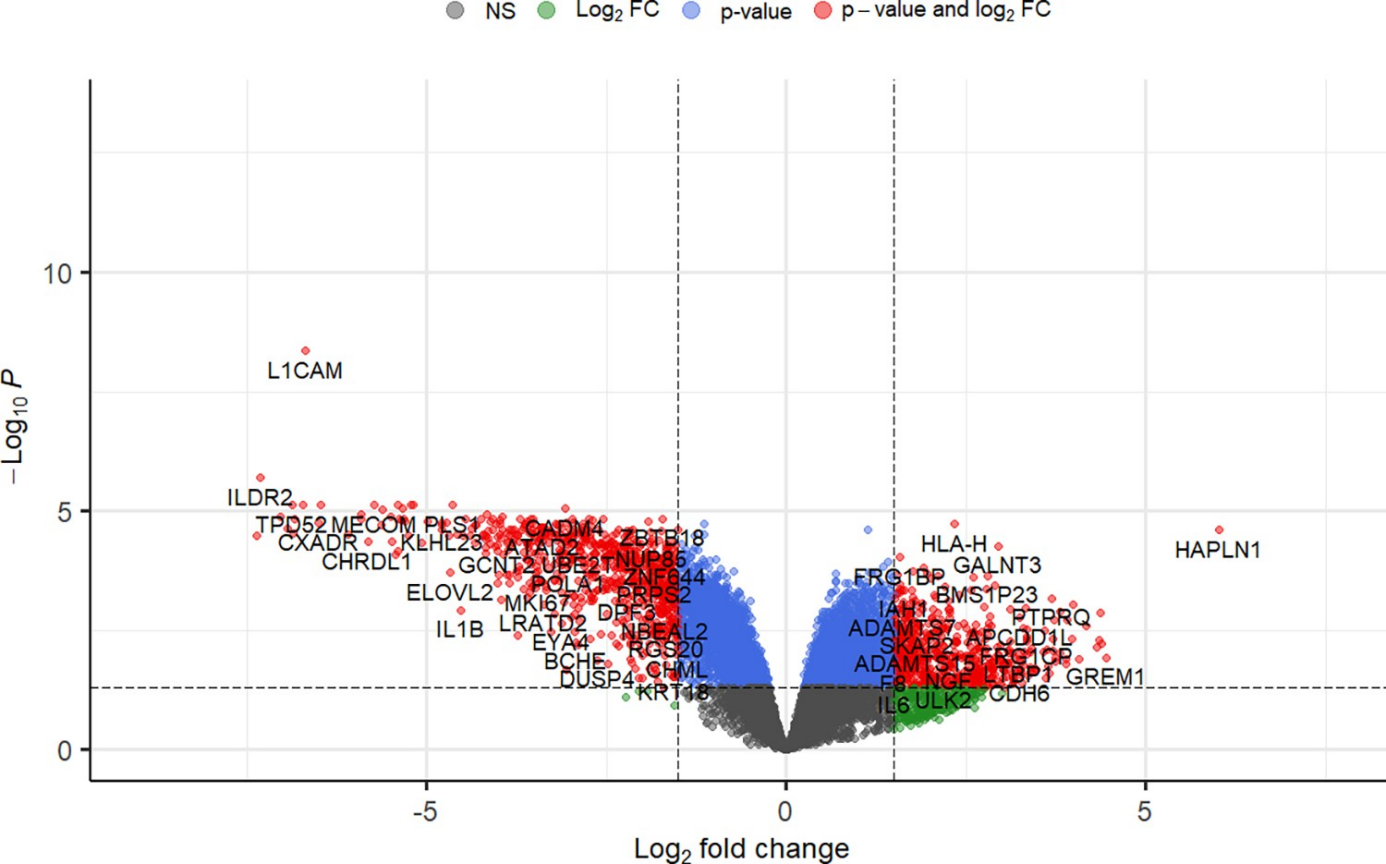
Volcano plot identifying differentially expressed genes between glaucomatous and normal Tenon’s ocular fibroblasts. In this figure grey and green dots represent genes that are not considered to be differentially expressed because the false discovery rate FDR>0.05, blue dots represent genes which are considered to be differentially expressed with a FDR<0.05 and |log_2_FC| ≤ 1.5 while red dots represent genes that are both significantly expressed and also have a high fold-change: FDR < 0.05 and |log_2_FC| > 1.5. In this work all genes with FDR < 0.05 and |log_2_FC| > 0.26 have been taken into account (all red genes and most blue genes) which amounts to 5817 genes of which 2664 genes were upregulated and 3153 genes were downregulated.

**Fig 2:**
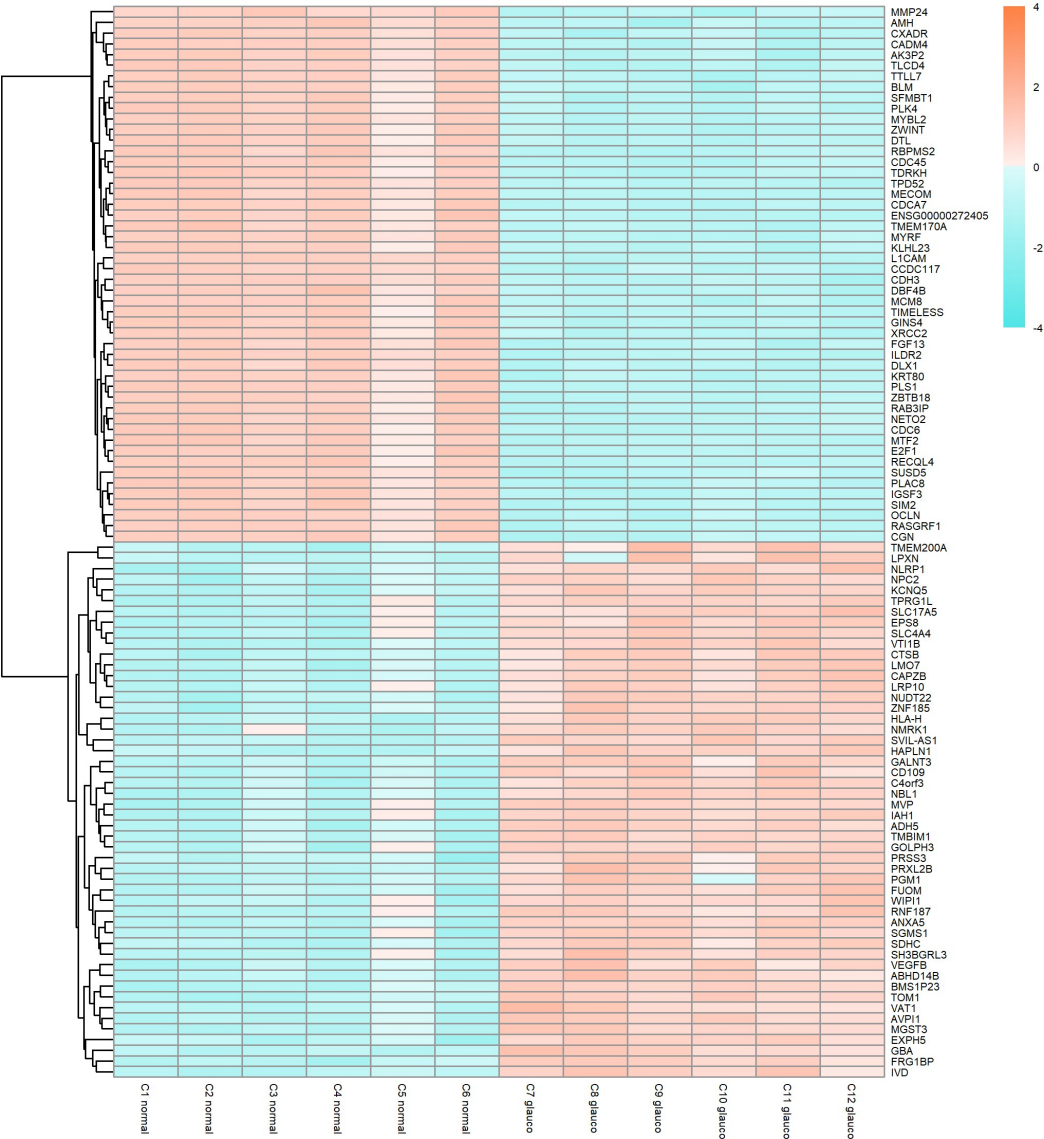
Heatmap showing the expression of 50 most upregulated and 50 most downregulated differentially expressed genes when ranked by lowest FDR (false discovery rate) in glaucomatous Tenon’s ocular fibroblasts when compared to normal Tenon’s ocular fibroblasts. The expression pattern of each gene is shown in log2cpm has been normalised to enable comparison between genes. Hierarchical clustering of genes has been applied to group genes by normalised gene expression pattern.

Fig 1 shows a volcano-plot of all genes in which thresholds were set at FDR = 0.05 and arbitrarily |log_2_FC| = 1.5 to show genes with highest fold-change. Of the total set of 11,227 expressed genes, 5817 were considered to be differentially expressed based on a false discovery rate FDR<0.05 and an absolute fold change |log_2_FC| > 0.26 (which corresponds to a fold change of at least 20%). Of these 5817 genes, 2664 genes were upregulated, and 3153 genes were downregulated. Housekeeping genes GAPDH and ACTB were not differentially expressed. Table 1 shows the top 25 upregulated and top 25 downregulated genes based on FDR and Fig 2 shows a heatmap of the normalised expression of top 50 upregulated and 50 downregulated genes with the lowest FDR. S1 Fig shows the 2 highest dimensions of a principal component analysis (PCA) which demonstrates good clustering of glaucomatous samples while normal samples show more spread, especially samples C3 and C5. A complete table containing all 11,227 genes that were expressed can be found in S1 Table.

**Table 1:** Top 25 differentially upregulated and top 25 downregulated genes in glaucomatous Tenon’s ocular fibroblasts when compared to normal Tenon’s ocular fibroblasts as ranked by false discovery rate (FDR).

Gene symbol	Gene name	log_2_FC	FDR
L1CAM (NCAM-L1)	L1 cell adhesion molecule	-6.69	4.38e-09
ILDR2 (C1orf32)	immunoglobulin like domain containing receptor 2	-7.32	1.99e-06
TPD52	tumor protein D52	-6.87	7.42e-06
MECOM (MDS1 EVI1)	MDS1 and EVI1 complex locus	-5.74	7.42e-06
PLS1	plastin 1	-4.63	7.42e-06
RASGRF1 (GRF1)	Ras protein specific guanine nucleotide releasing factor 1	-6.71	7.42e-06
MYBL2	MYB proto-oncogene like 2	-5.18	7.42e-06
DLX1	distal-less homeobox 1	-5.23	7.42e-06
CDH3	cadherin 3	-6.47	7.42e-06
MYRF (C11orf9)	myelin regulatory factor	-5.40	7.42e-06
CDCA7	cell division cycle associated 7	-5.33	8.93e-06
CADM4 (IGSF4C)	cell adhesion molecule 4	-3.08	8.93e-06
NETO2	neuropilin and tolloid like 2	-5.62	9.39e-06
IGSF3	immunoglobulin superfamily member 3	-5.91	1.16e-05
GINS4	GINS complex subunit 4	-4.16	1.16e-05
XRCC2	X-ray repair cross complementing 2	-3.95	1.32e-05
AK3P2	adenylate kinase 3 pseudogene 2	-7.03	1.32e-05
TLCD4 (TMEM56)	TLC domain containing 4	-5.50	1.32e-05
AMH	anti-Mullerian hormone	-4.07	1.46e-05
TTLL7	tubulin tyrosine ligase like 7	-2.55	1.47e-05
ENSG00000272405	ENSG00000272405	-5.31	1.47e-05
FGF13 (LINC00889)	fibroblast growth factor 13	-5.38	1.47e-05
SIM2 (SIM)	SIM bHLH transcription factor 2	-6.84	1.47e-05
MCM8 (C20orf154)	minichromos. maint.8 homologous recomb. repair factor	-3.55	1.47e-05
OCLN	occludin	-5.91	1.47e-05
HLA-H	major histocompatibility complex, class I, H (pseudogene)	2.34	1.89e-05
SVIL-AS1	SVIL antisense RNA 1	1.13	2.52e-05
HAPLN1 (CRTL1)	hyaluronan and proteoglycan link protein 1	6.02	2.52e-05
GALNT3	polypeptide N-acetylgalactosaminyltransferase 3	2.96	5.46e-05
FRG1BP (C20orf80)	FSHD region gene 1 family member B, pseudogene	1.58	9.25e-05
AVPI1	arginine vasopressin induced 1	1.41	1.14e-04
ADH5 (FDH)	alcohol dehydrogenase 5 (class III), chi polypeptide	1.35	1.46e-04
CD109	CD109 molecule	1.91	1.55e-04
KCNQ5	potassium voltage-gated channel subfamily Q member 5	1.76	1.87e-04
MGST3	microsomal glutathione S-transferase 3	1.18	1.92e-04
CAPZB	capping actin protein of muscle Z-line subunit beta	1.16	1.92e-04
TMBIM1	transmembrane BAX inhibitor motif containing 1	1.27	1.97e-04
GBA (GLUC)	glucosylceramidase beta	0.69	2.04e-04
LMO7 (FBXO20)	LIM domain 7	1.96	2.07e-04
ANXA5 (ENX2 ANX5)	annexin A5	1.44	2.13e-04
CTSB	cathepsin B	1.54	2.15e-04
BMS1P23	BMS1 pseudogene 23	2.81	2.28e-04
NMRK1 (C9orf95)	nicotinamide riboside kinase 1	2.10	2.36e-04
EXPH5	exophilin 5	1.48	2.36e-04
SLC4A4 (SLC4A5)	solute carrier family 4 member 4	2.60	2.41e-04
NUDT22	nudix hydrolase 22	1.01	2.44e-04
FUOM (C10orf125)	fucose mutarotase	2.01	2.55e-04
TOM1	target of myb1 membrane trafficking protein	0.69	2.84e-04
NPC2	NPC intracellular cholesterol transporter 2	1.00	2.90e-04
IVD	isovaleryl-CoA dehydrogenase	0.69	2.97e-04

For each gene the current and previously used symbols, gene name, log_2_FC (binary logarithm of fold change), p-value and adjusted p-value in the form of false discovery rate (FDR) are shown.

### Functional enrichment and pathway analysis

**Fig 3:**
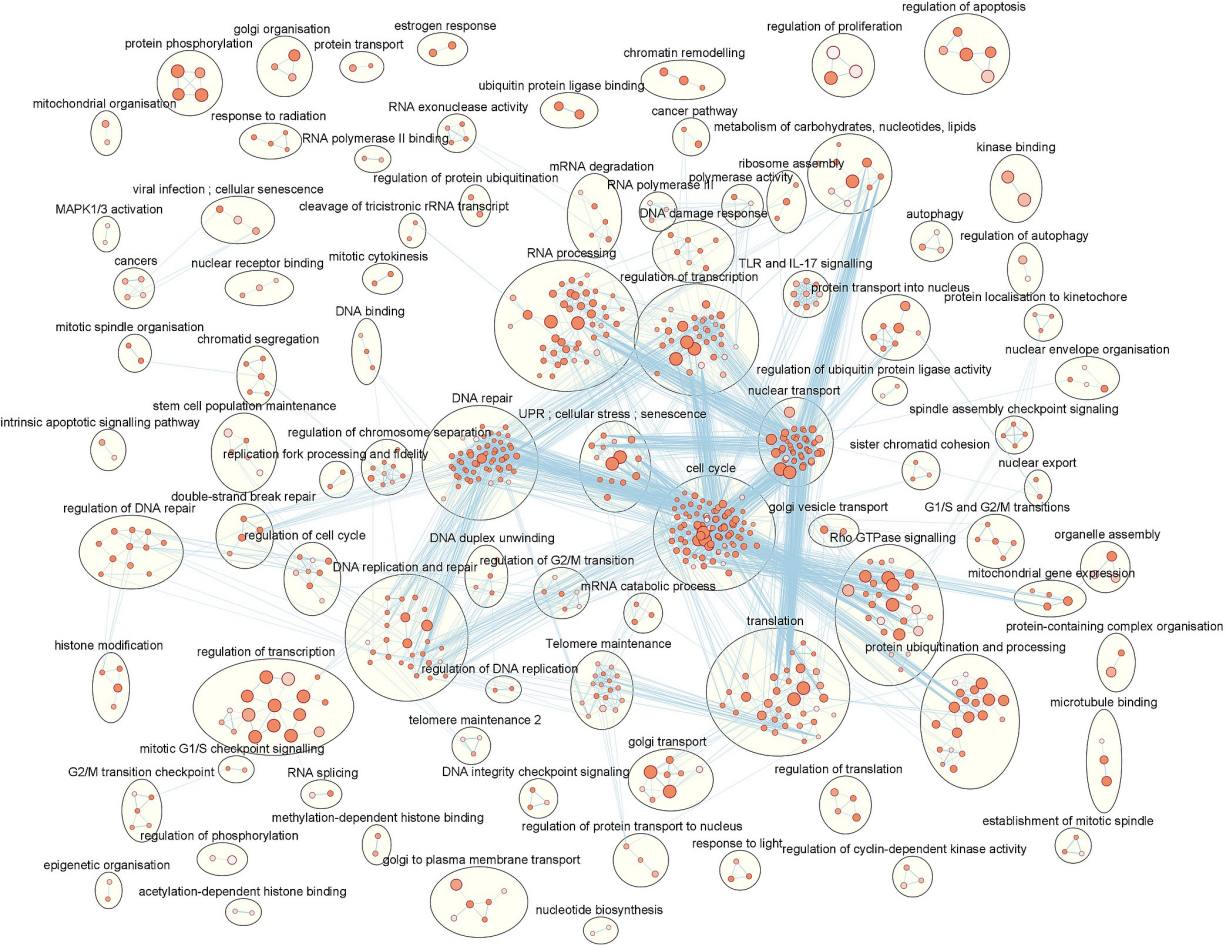
Clustered enrichment results showing key biological processes that are different between glaucomatous and normal Tenon’s ocular fibroblasts. The enrichment terms were obtained using an over-representation analysis (ORA) of a gene list of differentially expressed genes with false discovery rate FDR < 0.05 when comparing glaucomatous to normal Tenon’s capsule fibroblasts. Each node corresponds to an enrichment term in which the node intensity represents the FDR in a range 0.00 (darkest) to 0.01 (lightest) while the node size indicates the number of genes associated with a certain enrichment term. Blue lines connecting these nodes represent gene list overlap between enrichment terms above a threshold of 0.27. Clustered enrichment terms have been encircled and annotated.

Using functional enrichment tool Enrichr, overexpression analysis was performed on the list of 5817 differentially expressed genes to identify significantly altered pathways in pathway databases KEGG, WikiPathways, Reactome, MSigDB Hallmark genesets and enriched molecular functions (MF) and biological processes (BP) in Gene Ontology (GO). Enrichment terms were assumed to be significantly changed between glaucomatous and normal TFs if the false discovery rate FDR<0.01. This enrichment analysis yielded a total of 787 enriched pathways and GO terms containing the majority of DEGs. These enrichment results were clustered into 176 clusters of which 89 clusters containing only one term. Those clusters containing more than one term have been visualised in Fig 3. The largest cluster contained 93 terms all related to “cell cycle” pointing to a significant overlap and redundancy in terms. A list of the top 50 enrichment results (as ranked by FDR) can be found in Table 2. A full list of clusters, terms and genes per term can be found in S2 Table.

**Table 2:** Top 50 enriched pathways.

term	FDR
Cell Cycle (REAC:R-HSA-1640170)	9.18e-63
E2F Targets (MSIG:26)	4.92e-62
RNA Binding (GOMF:0003723)	5.15e-62
Cell Cycle, Mitotic (REAC:R-HSA-69278)	1.10e-52
Metabolism Of RNA (REAC:R-HSA-8953854)	1.87e-46
G2-M Checkpoint (MSIG:08)	1.48e-43
Processing Of Capped Intron-Containing Pre-mRNA (REAC:R-HSA-72203)	1.02e-34
M Phase (REAC:R-HSA-68886)	6.27e-33
Cell Cycle Checkpoints (REAC:R-HSA-69620)	1.26e-31
Myc Targets V1 (MSIG:27)	1.00e-29
DNA Metabolic Process (GOBP:0006259)	3.06e-29
Mitotic Prometaphase (REAC:R-HSA-68877)	3.51e-29
mRNA Splicing—Major Pathway (REAC:R-HSA-72163)	1.51e-26
Mitotic Metaphase And Anaphase (REAC:R-HSA-2555396)	3.02e-26
mRNA Splicing (REAC:R-HSA-72172)	5.12e-26
Mitotic Anaphase (REAC:R-HSA-68882)	5.52e-26
Resolution Of Sister Chromatid Cohesion (REAC:R-HSA-2500257)	1.63e-24
DNA Repair (REAC:R-HSA-73894)	1.55e-23
Gene Expression (Transcription) (REAC:R-HSA-74160)	2.00e-23
DNA Repair (GOBP:0006281)	2.01e-23
Separation Of Sister Chromatids (REAC:R-HSA-2467813)	2.15e-23
Metabolism Of Proteins (REAC:R-HSA-392499)	1.66e-22
Retinoblastoma gene in cancer (WIPA:WP2446)	1.87e-22
DNA Damage Response (GOBP:0006974)	5.22e-22
mRNA Splicing, Via Spliceosome (GOBP:0000398)	5.32e-21
Mitotic Spindle Checkpoint (REAC:R-HSA-69618)	6.65e-21
mRNA Processing (GOBP:0006397)	2.84e-20
Cellular Responses To Stress (REAC:R-HSA-2262752)	4.36e-20
Signaling By Rho GTPases, Miro GTPases And RHOBTB3 (REAC:R-HSA-9716542)	4.39e-20
Unattached Kinetochores Signal Amplification Via A MAD2.. (REAC:R-HSA-141444)	5.06e-20
RHO GTPases Activate Formins (REAC:R-HSA-5663220)	1.05e-19
mRNA Processing (WIPA:WP411)	1.51e-19
Cellular Responses To Stimuli (REAC:R-HSA-8953897)	3.64e-19
EML4 And NUDC In Mitotic Spindle Formation (REAC:R-HSA-9648025)	4.19e-19
Post-translational Protein Modification (REAC:R-HSA-597592)	4.19e-19
S Phase (REAC:R-HSA-69242)	5.63e-19
Signaling By Rho GTPases (REAC:R-HSA-194315)	7.58e-19
DNA Repair Pathways Full Network WP4946 (WIPA:WP4946)	3.72e-18
Transcriptional Regulation By TP53 (REAC:R-HSA-3700989)	4.69e-18
RNA Polymerase II Transcription (REAC:R-HSA-73857)	1.44e-17
RNA Splicing, Via Transesterification Reactions With Bulged Adenosine As.. (GOBP:0000377)	1.82e-17
Ribosome Biogenesis (GOBP:0042254)	7.31e-16
Mitotic G1 Phase And G1/S Transition (REAC:R-HSA-453279)	1.80e-15
Mitotic Spindle (MSIG:03)	3.28e-15
DNA IR-damage and cellular response via ATR (WIPA:WP4016)	3.60e-15
Epithelial Mesenchymal Transition (MSIG:29)	4.32e-15
G1/S Transition (REAC:R-HSA-69206)	5.51e-15
Mitotic G2-G2/M Phases (REAC:R-HSA-453274)	9.16e-15
Spliceosome (KEGG:hsa03040)	1.55e-14
G2/M Transition (REAC:R-HSA-69275)	3.63e-14

The top 50 enriched pathways from KEGG, Reactome (REAC), WikiPathways (WIPA), MSigDB Hallmark genesets (MSIG) and GO biological processes (GOBP) and molecular functions (GOMF) ranked by false discovery rate (FDR) in glaucomatous Tenon’s ocular fibroblasts.

### Glaucomatous Tenon’s ocular fibroblasts show signs of fibroblast to myofibroblast transdifferentiation

**Fig 4:**
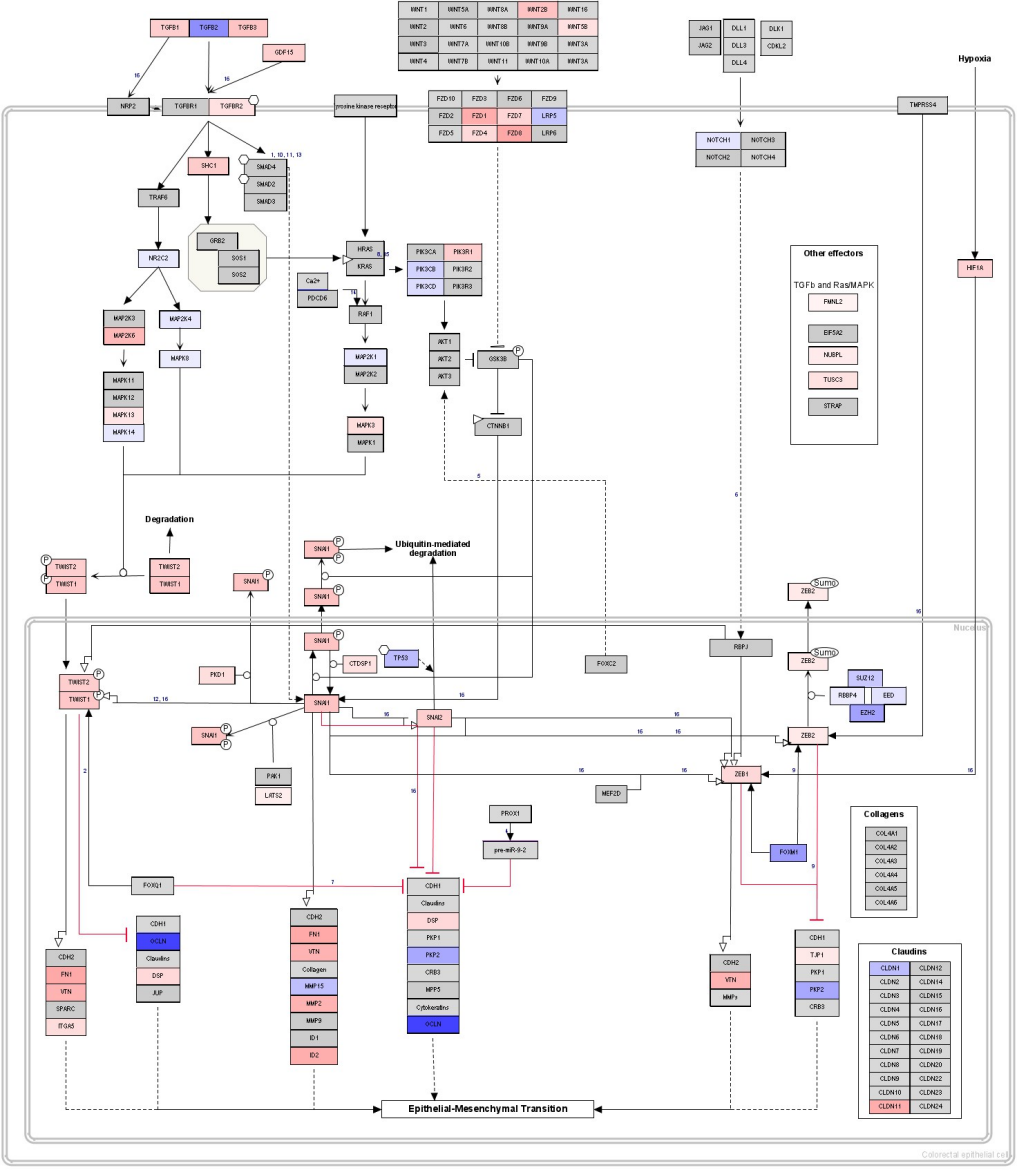
Pathway analysis showing myofibroblastic transition in glaucomatous Tenon’s ocular fibroblasts. Expression of genes in WikiPathway “Epithelial to mesenchymal transition in colorectal cancer” (WP4239). Differentially expressed genes in WikiPathway WP4239 have been annotated using package PathVisio. Red corresponds to upregulation in glaucomatous TF cells when compared to normal TF cells while blue corresponds to downregulation. Grey coloured genes are expressed but not differentially so. White coloured genes were not found to be expressed in our dataset or were filtered out due to low number of reads.

Enrichment analysis showed significant enrichment of several fibrosis and EMT related enrichment terms such as MSigDB hallmark gene set “Epithelial Mesenchymal Transition” (FDR = 4e-15), “Positive Regulation Of Epithelial To Mesenchymal Transition” (GO:0010718). Furthermore “Epithelial to mesenchymal transition in colorectal cancer” (WP4239, p = 0.06) has a gene overlap of 56/160 and the annotated pathway shows several key genes involved in the processes of fibrosis as shown in Fig 4. Some key upregulated genes in this pathway are TGF-β1 and its receptor TGFBR2, transcription factors TWIST1, TWIST2, ZEB1, ZEB2 and transcriptional repressors SNAI1, SNAI2. Furthermore, effector genes FN1 and several collagens were upregulated which is in keeping with a profibrotic status. Not shown in Fig 4 is key fibrotic marker ACTA2 which was also upregulated (log_2_FC = 2.9).

### Increased synthesis and remodelling of the extracellular matrix in glaucomatous Tenon’s ocular fibroblasts

The enrichment analysis resulted in several terms associated with synthesis and remodelling of the extracellular matrix such as “Crosslinking Of Collagen Fibrils R-HSA-2243919” and “Regulation Of Extracellular Matrix Organization (GO:1903053)”, see S2 Fig. The expression of collagen species COL1A1, COL6, COL8, COL12 and possibly COL5A1 (FDR = 0.053) was upregulated in glaucomatous TF cells when compared to normal TF cells. Expression of laminin subunits LAMA4, LAMC1, LAMC2 was also upregulated. Furthermore, ECM components including VCAN (versican), FN1 (fibronectin), FBN1 (fibrillin-1), VTN (vitronectin) were upregulated in glaucomatous TF cells. ELN (elastin) was not differentially expressed. Latent transforming growth factor binding proteins LTBP1, LTBP2, LTBP3 were all strongly upregulated in glaucomatous TF cells and so was THBS1 (thrombospondin). With respect to collagen synthesis, ECM enzymes including LOX, LOXL1, LOXL3, LOXL4, PXDN, P4HA1, P4HA2, PLOD1, PLOD3 were all upregulated. Considering the matrix metallopeptidase family, ADAM12, ADAM10, ADAM23 were upregulated and so were MMP2 (log_2_FC = 2.4), MMP14 and MMP17 while MMP15 and MMP24 were downregulated. Conversely, metallopeptidase inhibitors TIMP1, TIMP2, TIMP3 were also strongly upregulated, for instance TIMP1 showed log_2_FC = 3.1 corresponding to an 8-fold increase with respect to normal TF cells.

### Autophagy is upregulated in glaucomatous Tenon’s ocular fibroblasts

**Fig 5:**
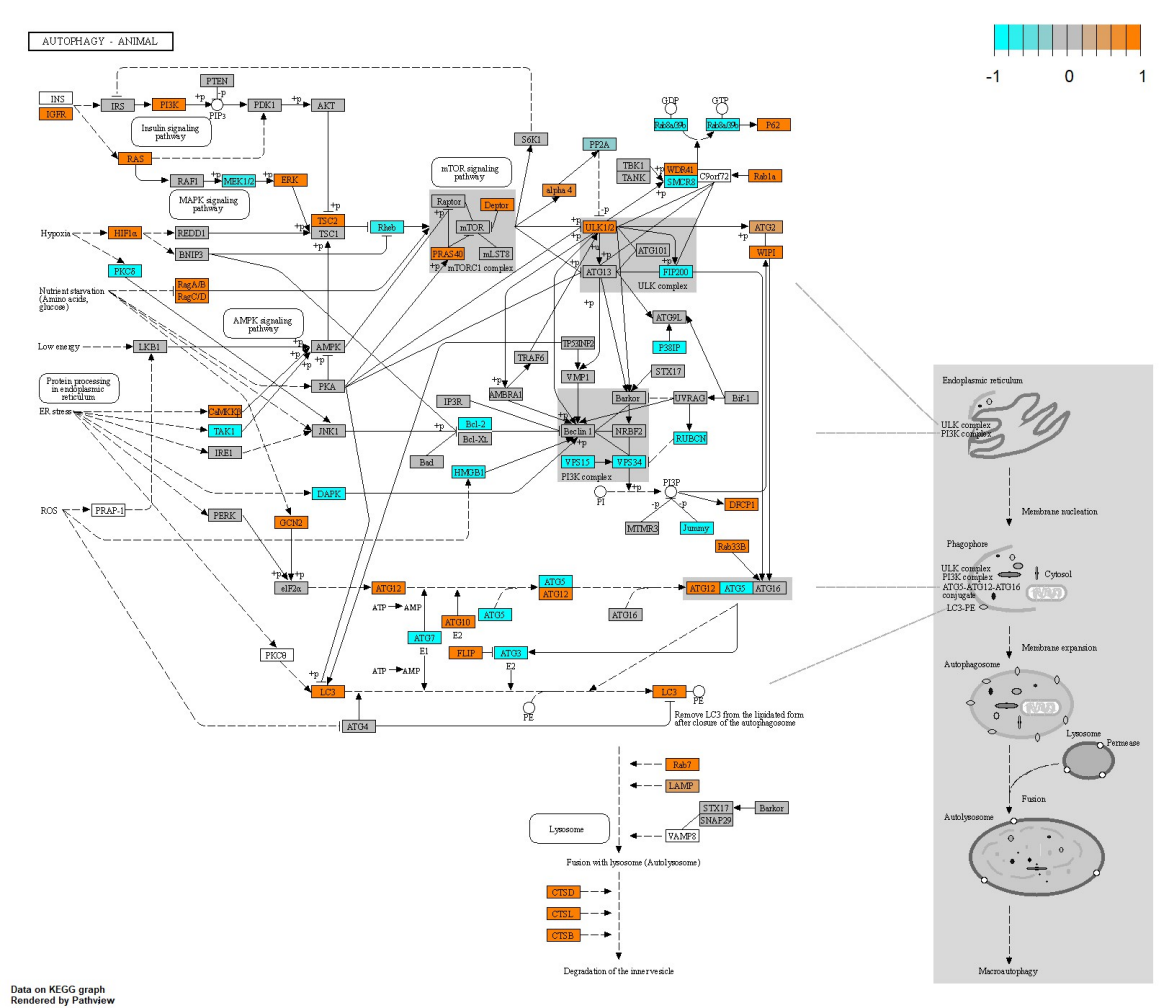
Pathway analysis showing autophagy in glaucomatous Tenon’s ocular fibroblasts when compared to normal fibroblasts. The autophagy transcriptome in glaucomatous Tenon’s ocular fibroblasts by annotation of genes in KEGG pathway hsa04140 named “autophagy—animal” using R package Pathview. To enhance visibility, gene expression was normalised to a range of [–1,1] in which orange indicates upregulation and cyan indicates downregulation. Grey coloured genes are expressed but not differentially so. White coloured genes were not found to be expressed at sufficient levels in our dataset.

Enrichment analysis showed significant changes in autophagy in glaucomatous TFs compared to normal TFs as indicated by enriched Reactome pathways “Autophagy” (R-HSA-9612973, FDR = 1.8e-3), “Selective Autophagy” (R-HSA-9663891, FDR = 8.8e-3) and “Macroautophagy” (R-HSA-1632852) and GO biological processes “Macroautophagy” (GO:0016236) and “Regulation Of Macroautophagy” (GO:0016241). Some key genes involved in autophagy are shown in a heatmap in S3 Fig and in the annotated KEGG pathway hsa04140 in Fig 5.

Several genes related to LC3 formation were upregulated such as MAP1LC3A, MAP1LC3B and GABARAPL1. Of the ATG (autophagy related) gene family, ATG2B, ATG10, ATG12, ATG14 were upregulated and ATG3, ATG5, ATG7 were moderately downregulated. Furthermore, LAMP1 and SQSTM1 (p62) were upregulated. While autophagy did appear to be upregulated, this was not clear for mitophagy. Several mitophagy-related enrichment results were identified such as “PINK1-PRKN Mediated Mitophagy” (R-HSA-5205685) but further inspection demonstrated that there was no change in expression of PINK1 and there was hardly any expression of PRKN and in fact this gene was filtered out due to low count numbers.

### Mitochondrial fission is upregulated in glaucomatous Tenon’s ocular fibroblasts

**Fig 6:**
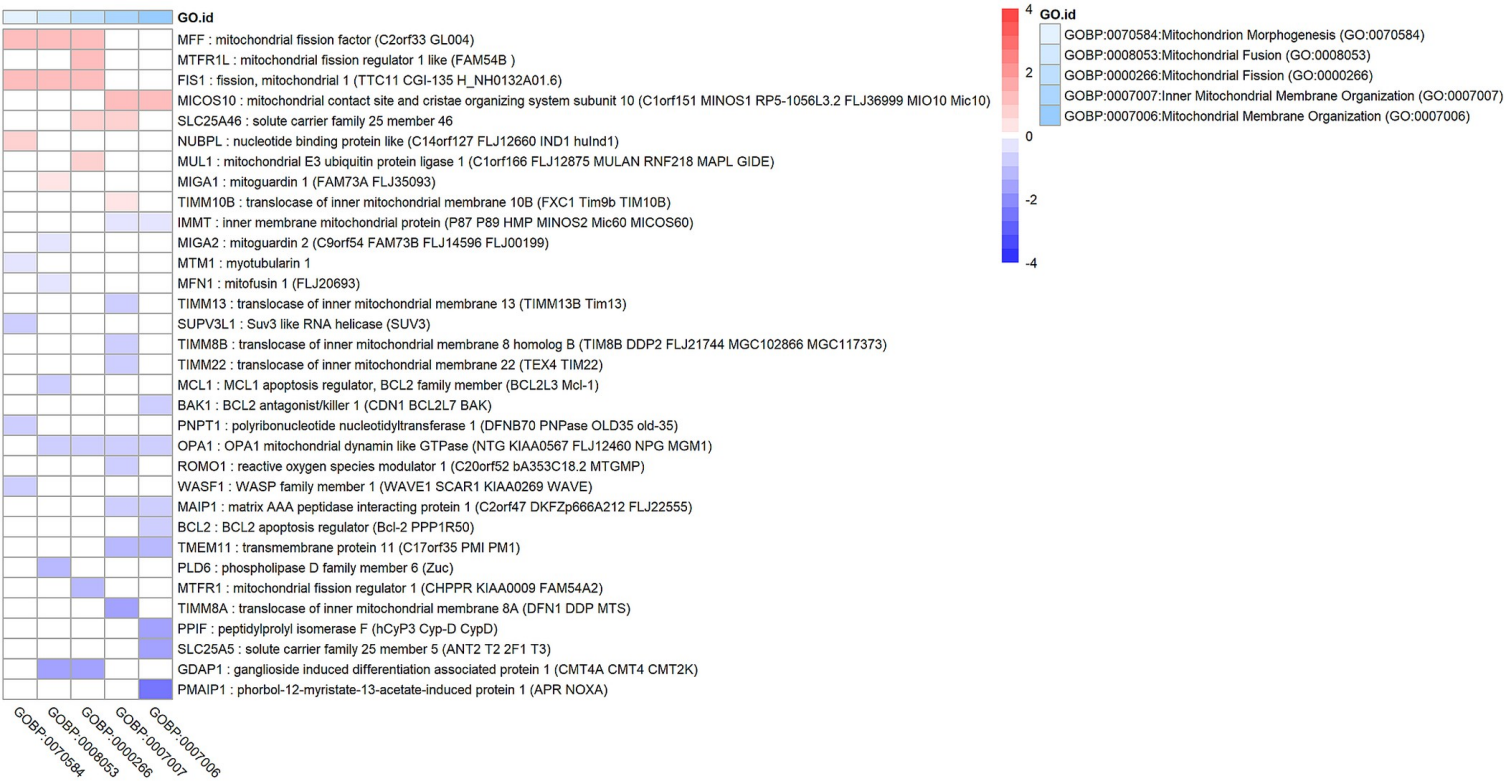
Genes and enrichment terms associated with mitochondrial fission and fusion in glaucomatous Tenon’s ocular fibroblasts when compared to normal Tenon’s ocular fibroblasts. This heatmap shows genes with a false discovery rate FDR < 0.05 that are associated with key enrichment terms related to mitochondrial fission and fusion. Heatmap colours correspond to fold change (in log_2_FC) which has been limited to a range of [–4,4] to improve visibility.

Several biological processes involved in mitochondrial fission and fusion were significantly altered such as Mitochondrion Morphogenesis (GO:0070584) and Mitochondrion Organization (GO:0007005). The differentially expressed genes involved in these biological processes and other related processes are shown in Fig 6. Several key genes associated with mitochondrial fission were upregulated such as FIS1, MFF and several genes involved in mitochondrial fusion were downregulated such as MFN1 and OPA1 which suggests a functional enhancement of mitochondrial fission.

### Oxidative stress is increased in glaucomatous Tenon’s ocular fibroblasts

**Fig 7:**
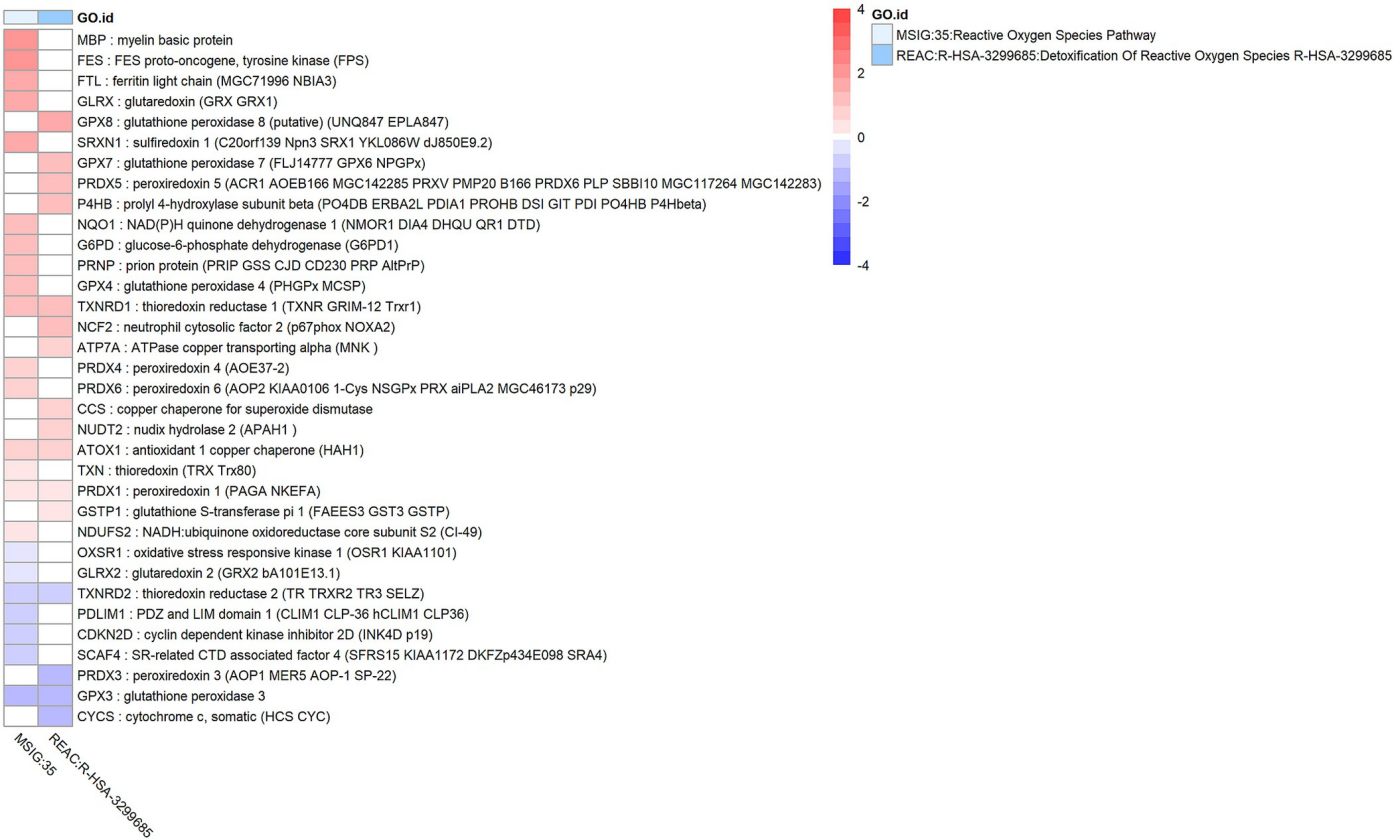
Genes and enrichment terms associated with oxidative stress. Heatmap showing genes with false discovery rate FDR < 0.05 that are associated with key enrichment terms related to oxidative stress and the antioxidant system. Heatmap colours correspond to fold change (in log_2_FC) between glaucomatous and normal Tenon’s ocular fibroblasts which has been limited to a range of [–4,4] to improve visibility.

Oxidative stress was identified in our enrichment results, for instance “Cellular Response To Oxidative Stress (GO:0034599)” and MSigDB hallmark set “Reactive Oxygen Species Pathway”. The genes associated with some key enrichment terms are presented as a heatmap in Fig 7. Several antioxidant enzymes were upregulated including GLRX (glutaredoxin), SRXN1 (sulfiredoxin), TXNRD1 and glutathione peroxidases GPX8, GPX7, GPX4 while GPX3 was downregulated in glaucomatous TF cells. Peroxiredoxins PRDX1, PRDX4, PRDX5, PRDX6 were upregulated in glaucomatous TF cells while PRDX3 was downregulated. One component of glutathione S-transferase, namely GSTP1, was upregulated. Key regulator of antioxidant enzymes NFE2L2, also known as NRF2, was upregulated by a factor of two (log2FC = 0.94, FDR = 3e-3) in glaucomatous TF cells. There was no difference in expression of SOD (superoxide dismutase) enzymes between glaucomatous and normal TF cells. Overall, the antioxidant system and especially the glutathione subsystem was clearly upregulated in glaucomatous TF cells when compared to normal TF cells.

### Glaucomatous Tenon’s ocular fibroblasts show cell cycle arrest and senescence

**Fig 8:**
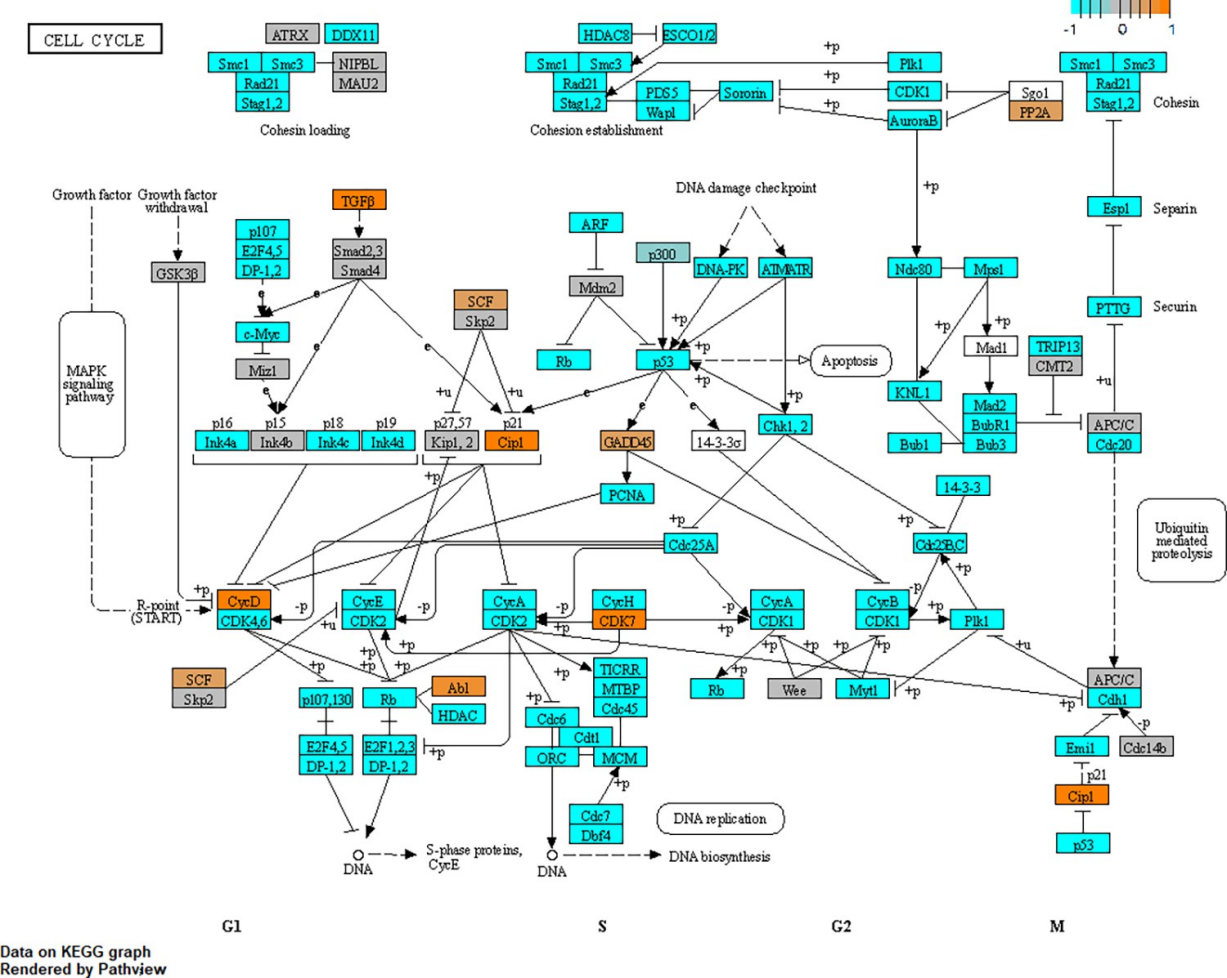
Pathway analysis showing cell cycle alterations in glaucomatous Tenon’s ocular fibroblasts. Alterations in cell cycle have been shown by annotation of genes in KEGG pathway hsa04110 named “cell cycle” using R package Pathview. To enhance visibility, gene expression has been normalised to a range of [–1,1] in which orange indicates upregulation and cyan indicates downregulation. Grey coloured genes are expressed but not differentially so. White coloured genes were not found to be expressed at sufficient levels in our dataset.

Enrichment cluster “cell cycle” was the largest cluster in our enrichment results and contained 93 enrichment terms all related to the cell cycle such as KEGG “cell cycle” hsa04110 (FDR = 4e-14) and “Cell Cycle R-HSA-1640170” in Reactome, see S2 Table, indicating that there are differences in proliferation and cell cycle between glaucomatous and normal TFs. To illustrate key differences in cell cycle gene expression, KEGG diagram hsa04110 was annotated using Pathview (see Fig 8), demonstrating clear differences in gene expression between glaucomatous and normal TFs. Further inspection of Cyclins and Cdk inhibitor genes in glaucomatous versus normal TFs showed that CCND1 was upregulated and CCNB2, CCNE2, CCNB1, CCNA2 were all highly downregulated (log2FC around -3.5) in glaucomatous TFs, indicating that these cells were most likely in the G1 phase. Furthermore, CCNG1 was upregulated and Cdk inhibitor CDKN1A (p21, Cip1) was upregulated while all other Cdk inhibitors were either downregulated or not differentially expressed. Cyclin expression is in part regulated by transcription regulators of the E2F family. Within this family, E2F1, E2F3, E2F4 were all downregulated and so were their inhibitors RB1, RBL1 and RBL2. In addition to cell cycle arrest, glaucomatous TFs also showed signs of a senescence-associated secretory phenotype including upregulation of TGF-β1 (TGFB1, log2FC = 1.5, FDR = 0.006), IL6 (log2FC = 1.5, FDR = 0.045), IGFBP2, IGFBP3, IGFBP4, IGFBP6. SERPINE1 (PAI-1) was also upregulated but with FDR = 0.06. All VEGF species VEGFA, VEGFB, VEGFC were significantly upregulated. In summary, the glaucomatous TF cells show cell cycle arrest and a senescence-associated secretory phenotype. With respect to the TGF-β family it is interesting to note that while TGF-β1 (TGFB1) and TGF-β3 (TGFB3) were both upregulated, expression of TGF-β2 (TGFB2) was strongly downregulated (log2FC = -3.5, FDR = 2e-5) in glaucomatous TF cells when compared to normal cells.

### Upregulation of the unfolded protein response in glaucomatous Tenon’s ocular fibroblasts

**Fig 9:**
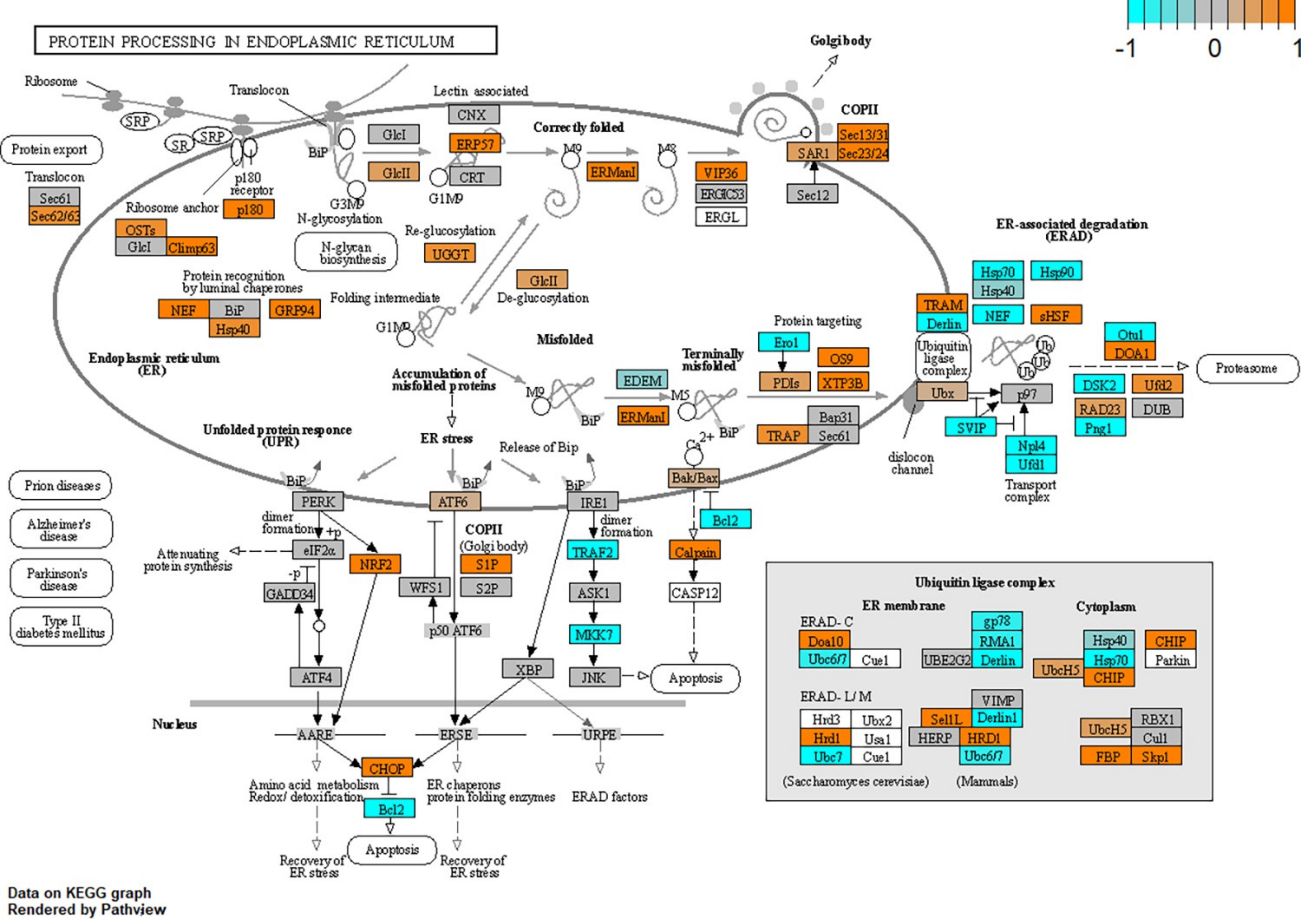
Pathway analysis showing alterations in the unfolded protein response and ER stress. Changes in the unfolded protein response and ER stress in Tenon’s ocular fibroblasts have been shown by annotation of genes in KEGG pathway hsa04141 “protein processing in endoplasmic reticulum” which also contains the Unfolded Protein Response (UPR). Annotation has been achieved using R package Pathview. To enhance visibility, gene expression has been normalised to a range of [–1,1] in which orange indicates upregulation and cyan indicates downregulation. Grey coloured genes are expressed but not differentially so. White coloured genes were not found to be expressed at sufficient levels in our dataset.

The unfolded protein response (UPR) appeared in several enrichment terms within cluster “UPR; cellular stress; senescence” such as “XBP1(S) Activates Chaperone Genes” (R-HSA-381038), “Unfolded Protein Response (UPR)” (R-HSA-381119), “ERAD Pathway” (GO:0036503) and “ATF4 Activates Genes In Response To Endoplasmic Reticulum Stress R-HSA-380994”. Further inspection and pathway analysis showed increased expression of DDIT3 (CHOP), see Fig 9 and the gene-enrichment heatmap in S4 Fig. There are three UPR branches that may be activated and that start by dissociation of HSPA5 (BiP) from EIF2AK3 (PERK), ATF6 or ERN1 (IRE1α). HSPA5 itself was not differentially expressed. EIF2AK3 (PERK) was also not differentially expressed but a paralog of this gene, EIF2AK4, was upregulated possibly indicating activation of this UPR branch. ATF6B was moderately upregulated. ERN1 (IRE1α) was not differentially expressed but its associated gene XBP1 was upregulated albeit with an FDR = 0.06. In summary the UPR is upregulated or at least altered in glaucomatous TFs when compared to normal TF cells.

### Glaucomatous Tenon’s ocular fibroblasts show altered apoptotic pathways

**Fig 10:**
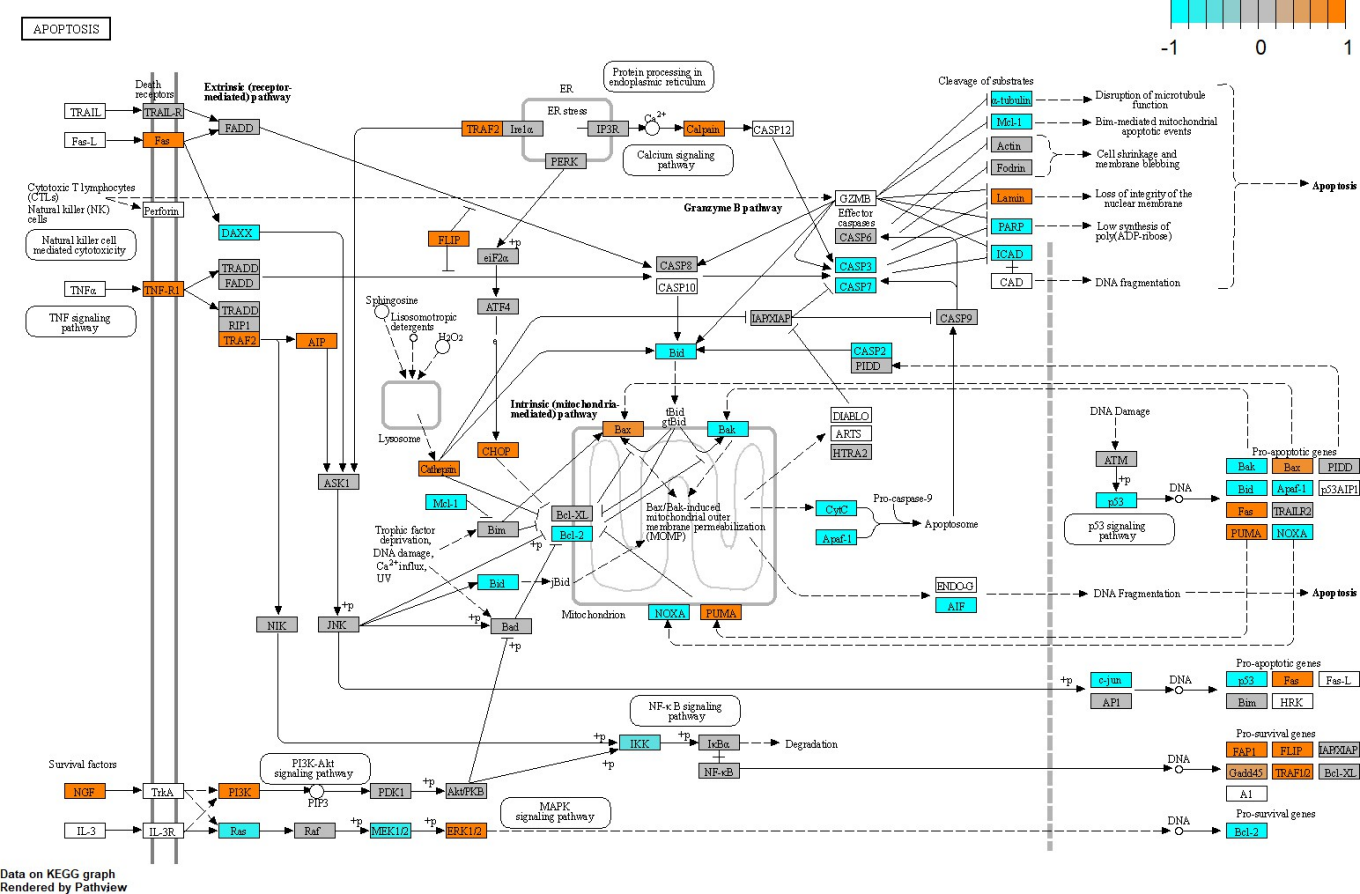
Pathway analysis showing apoptosis related gene expression in glaucomatous Tenon’s ocular fibroblasts compared to normal Tenon’s ocular fibroblasts. The pathway analysis is performed by annotation of genes in KEGG pathway hsa04210 “apoptosis” using R package Pathview. To enhance visibility, gene expression has been normalised to a range of [–1,1] in which orange indicates upregulation and cyan corresponds to downregulation. Grey coloured genes are expressed but not differentially so. White coloured genes were not found to be expressed at sufficient levels in our dataset.

Enrichment analysis resulted in two clusters involved in apoptosis, “regulation of apoptosis” and “intrinsic apoptotic signalling pathway” and several unclustered terms. The intrinsic apoptotic pathway showed both pro-apoptotic and anti-apoptotic changes in glaucomatous TF cells when compared to normal cells, see the annotated KEGG pathway hsa04210 in Fig 10. BH3-only sensitizers PMAIP1 (also known as NOXA, log_2_FC = -2.7, FDR = 2e-4) and BMF were downregulated while BAD was upregulated. Pro-survival genes BCL2 (log_2_FC = -0.94) and MCL1 were downregulated while expression of BCL2L1 (BCL-XL) was unchanged and expression of pro-survival gene BCL2L2 (also known as BCL-W) was upregulated. BH3-only activators BCL2L11 (BIM) and BID were downregulated while BBC3 (PUMA) was upregulated. Effector BAX was upregulated while BAK1 was downregulated. As mentioned before, DDIT3 (CHOP) was upregulated in glaucomatous tissue, linking the UPR to apoptosis. The extrinsic apoptotic pathway seemed upregulated in glaucomatous TF cells and showed upregulation of several genes coding for death receptors such as FAS and TNFRSF1A (TNF-R1). Transcription factor TP53 (P53) was downregulated by a factor 4 (log_2_FC = -2.1) and so was the expression of some of its transcriptional targets such as PMAIP1 but on the other hand expression of other TP53-regulated genes BBC3 (PUMA), BAX and FAS was upregulated. Executioner caspases CASP3 and CASP7 were downregulated while expression of Caspase inhibitor XIAP was unchanged.

### Changes in inflammation in glaucomatous Tenon’s fibroblasts

Several enrichment results pointed to differences in inflammation between GTF and NTF cells such as Reactome pathways “MyD88-independent TLR4 Cascade” (R-HSA-166166), “TRAF6 Mediated Induction Of NFkB And MAP Kinases Upon TLR7/8 Or 9 Activation” (R-HSA-975138),”Interleukin-17 Signaling” (R-HSA-448424), “Interleukin-6 Signaling” (R-HSA-1059683) and “Activation Of NF-kappaB In B Cells” (R-HSA-1169091). Further inspection on gene level identified upregulation of cytokine IL6 (log_2_FC = 1.5, FDR = 0.04) and downregulation of IL1B (IL-1β) (log_2_FC = -4.5, FDR = 0.001). Of the cytokine receptors, IL31RA was strongly downregulated (log_2_FC = -4.1), IL17RA was upregulated and so was IL6 receptor associated gene IL6ST (log_2_FC = 1.4). Toll-like receptor 4 (TLR4) was upregulated (log_2_FC = 2.1, FDR = 0.03). Several members of the TNF receptor superfamily were upregulated, notably TNFRSF1A and TNFRSF1B. As noted earlier, expression of TGF-β1 was upregulated (TGFB1, log_2_FC = 1.5, FDR = 0.006) while expression of TGF-β2 was downregulated (log_2_FC = -3.5, FDR = 2e-5) in glaucomatous TF cells when compared to normal cells.

## Discussion

In this study we performed a genome-wide hypothesis-independent RNA sequencing analysis to compare Tenon’s ocular fibroblasts from glaucoma (POAG) patients to those originating from non-glaucomatous patients. The large number of differentially expressed genes indicates that Tenon’s ocular fibroblasts from glaucomatous patients have undergone dramatic changes when compared to those from non-glaucomatous patients. Enrichment analysis revealed fibroblast to myofibroblast transdifferentiation and changes in mitochondrial organisation, autophagy, oxidative stress, ECM remodelling, inflammatory state, cell cycle arrest and senescence, unfolded protein response and apoptosis. These themes will be discussed in more detail below.

When comparing TF cells originating from glaucoma patients with those from normal patients, we found that glaucomatous TF cells showed upregulation of genes associated with a myofibroblast cell type such as ACTA2 (α-SMA), ZEB1, ZEB2, SNAI1 (SNAIL) and SNAI2 (SLUG) suggesting fibroblast to myofibroblast transdifferentiation. Myofibroblasts are contractile, ECM-producing cells that normally appear transiently during normal wound healing but become persistent in pathological fibrotic conditions such as systemic sclerosis [31], idiopathic pulmonary fibrosis [32] and liver fibrosis [33]. Myofibroblasts may originate from epithelial cells (via a process called EMT), endothelial cells (via EndMT), regular fibroblasts and other cells [34, 35]. Myofibroblasts and corresponding biological processes such as EMT have been reported to play a role in the context of glaucoma, for instance in retinal pigment epithelial cells [36], lamina cribrosa cells [37] and trabecular meshwork cells [38, 39]. In keeping with our results, Tenon’s ocular fibroblasts from glaucomatous patients were reported to show signs of a myofibroblast phenotype by determining differential expression of a predefined panel of 10 genes [16].

One mechanism by which fibroblasts may transdifferentiate into myofibroblasts is via exposure to POAG-related extracellular signal molecules such as TGF-β2 which is upregulated in the aqueous humour (AH) of POAG patients [8, 40]. It is plausible that AH containing POAG-associated ROS and cytokines can reach Tenon’s capsule and therefore TFs by drainage via the subconjunctival space and/or lymphatic vessels of the conjunctiva [41]. Also, TGF-β1 can cause fibroblast to myofibroblast transdifferentiation [42, 43] and may play an autocrine role in our experiment via a positive feedback mechanism because TGF-β1 (TGFB1) was upregulated in glaucomatous TF cells when compared to normal cells in our results. Another mechanism by which fibroblast to myofibroblast transdifferentiation may occur is in response to altered mechanical forces exerted onto the cell [44, 45]. It is not clear if such a mechanism has played a role in GTFs when compared to NTFs. The use of preservative benzalkonium chloride which is often present in topical glaucoma drugs has been demonstrated to cause TF fibroblast to myofibroblast transdifferentiation at lower concentrations than used in actual eyedrops [46] suggesting this could be a factor contributing to the fibroblast to myofibroblast transdifferentiation we observed in our results. Furthermore, exposure to reactive oxygen species (ROS) may also promote fibroblast to myofibroblast transdifferentiation [47, 48].

Overall, the increased expression of myofibroblast markers in glaucomatous TF tissue may be explained by exposure to POAG-related cytokines, growth factors and reactive oxygen species (ROS) in combination with ingredients of glaucoma drugs. Several aspects of fibrosis and myofibroblasts such as oxidative stress, autophagy, mitochondrial changes, extracellular matrix remodelling, inflammation, apoptosis and senescence will be discussed in more detail.

Our results showed increased expression of several ECM components such as collagen species, FN1, FBN1, FBN2 and others together with upregulation of ECM-modifying enzymes such as lysyl oxidases, prolyl hydroxylases, peroxidasin in glaucomatous TF cells when compared to normal TF cells. Upregulation of collagens is a key feature of many fibrotic conditions and especially the expression of COL1, COL3 and COL5 is increased in fibrosis [45]. Similar to our results, upregulation of MMP2 and MMP14 together with upregulation of TIMP1 has been shown in liver fibrosis [49]. Upregulation of TIMP2 and other TIMP species has been found in several forms of glaucoma as well [50–52] and will promote ECM accumulation. In keeping with our results, metallopeptidase ADAM12 has also been reported to be upregulated in glaucomatous lamina cibrosa cells [53] and is also associated with liver-fibrosis [54]. The expression of ADAM12 is regulated by TGF-β1 [55] and expression was shown to be upregulated *in vitro* in human trabecular meshwork cells after treatment with TGF-β1 [56]. Several lysyl oxidases were upregulated in our results which has also been shown in other myofibroblasts [34]. Upregulation of LOXL1 was also observed *in vitro* in Tenon’s ocular fibroblasts treated with TGF-β1 [57]. Since TGF-β1 was also upregulated in our data, LOXL1 expression may have been increased as a result of autocrine signalling via TGF-β1. Overall, glaucomatous TF cells show higher expression of structural ECM components while restraining the activity of matrix metalloproteases by increased expression of TIMPs which is expected to result in increased deposition of extracellular matrix proteins which highly correlates with fibrosis and fibroblast to myofibroblast transdifferentiation [45].

We observed differences in several autophagy related genes when comparing glaucomatous to normal TF cells. Several LC3 components of the MAP1LC3 family were upregulated but on the other hand ATG5 and ATG7 were moderately downregulated with a log_2_FC around -0.5. To assess differences in autophagy it would also have been interesting to determine the phosphorylation state of certain autophagy-related proteins such as LC3 which was beyond the scope of this study. Autophagy has been reported to be associated with several fibrotic conditions and can either promote or reduce fibrosis depending on cell type and cell state [58–60]. Furthermore, autophagy also plays a role in glaucoma. In POAG, autophagy in the trabecular meshwork has been shown to be dysregulated via the mTOR pathway [61] and autophagy may contribute to TM cell death [62] while autophagy deficiency protects against increased IOP and neurodegeneration in a mouse model [63, 64]. In pseudoexfoliation glaucoma dysfunctional autophagy may also play a causative role [65]. Overall, it appears that the observed differences in autophagy between glaucomatous and normal TF cells may be part of a fibrotic response.

Our results showed increased mitochondrial fission in glaucomatous TFs when compared to normal TFs. In several cell types mitochondrial fission has been reported to be associated with fibrosis. In corneal myofibroblast differentiation, mitochondrial fission was observed and in fact proved to be necessary to transdifferentiate corneal fibroblasts into myofibroblasts [66]. Mitochondrial fission and fragmentation were also reported in interstitial fibroblasts originating from fibrotic kidneys [67]. In myofibroblasts obtained from fibrotic lung tissue, mitochondrial fission was also found to be upregulated, presumably to meet higher energy demands [68]. With respect to glaucoma, increased mitochondrial fission has been observed after elevation of intraocular pressure in a mouse model [69]. Mitochondrial dysfunction and mitochondrial changes are strongly associated with glaucoma [70, 71] and with fibrotic conditions in general [72]. Mitochondrial changes have also been associated with exposure to glaucoma drug preservatives notably benzalkonium chloride (BAC). BAC has been reported to be causing mitochondrial dysfunction resulting in reduced ATP synthesis, production of reactive oxygen species [9, 10, 73] and mitochondrial fragmentation, although it should be noted that the latter observation was demonstrated in yeast [74].

The observed mitochondrial fission in our results is probably part of a fibrotic state which itself may have been caused by a combination of POAG pathology and glaucoma drug ingredients. The fact that mitochondrial changes have also been observed in animal models without BAC [69] suggests that at least POAG itself plays a role here.

There was upregulation of several key antioxidant enzymes and their primary regulator NFE2L2 (NRF2) in glaucomatous TF cells when compared to normal cells indicating that glaucomatous TF cells may have been subjected to increased oxidative stress levels compared to normal cells. In previous work our group demonstrated increased oxidative stress levels in glaucomatous Tenon’s ocular fibroblasts using the CellROX green assay [19]. Oxidative stress has also been reported to be associated with several fibrotic conditions such as pulmonary fibrosis [75]. One possible source of oxidative stress is BAC which has been shown to cause an increase in reactive oxygen species (ROS) and subsequent stress-induced premature senescence [10, 76] but oxidative stress is also a key feature of POAG and other forms of glaucoma [77, 78] and can be induced in a BAC-free mouse model using microbead occlusion [79]. Several transcriptional targets of NFE2L2 were upregulated in our results such as GPX4, PRDX6 and TXNRD1 [80] suggesting that NFE2L2 has not only been expressed at a higher level but also has a higher level of activation (i.e. phosphorylation), possibly via the PI3K/Akt pathway as described in [79]. NFE2L2 (NRF2) has been linked to glaucoma [81] and TGF-β2 [82] and may be a useful drug target to improve the outcome of glaucoma filtration surgery by reducing bleb fibrosis [83]. Reactive oxygen species have been shown to liberate latent TGF-β1 from the Large Latent Complex (consisting of TGF-β1, LAP and LTBP) by conformational change of LAP-beta1 [84] and possibly by other mechanisms as well [85].

When comparing glaucomatous TF cells to normal TF cells our results showed cell cycle arrest and upregulation of Cdk inhibitor protein CDKN1A (p21). CDKN1A binds to G1-Cdk thus inhibiting cell cycle progression at G1 [86]. It is interesting to note that CDKN1A is associated with POAG [87] and also with certain fibrotic conditions such as idiopathic pulmonary fibrosis [88]. Furthermore, our results showed signs of a senescence-associated secretory phenotype (SASP) as described in [89]. Senescence and cell cycle arrest are associated with glaucoma [90] as demonstrated by increased senescence in the outflow facility of POAG patients [91]. Upregulation of CDKN1A has also been linked to exposure to benzalkonium chloride, at least in human lung epithelial cells [92]. Cell cycle arrest can be induced by TGF-β2 (TGFB2) and is in this case associated with upregulation of CDKN2B [82] but in our results CDKN2B was not differentially expressed. In a model of pulmonary fibrosis, upregulation of CDKN1A and cell cycle arrest was also reported together with myofibroblast activation, telomere shortening and release of inflammatory cytokines pointing to a both senescent and fibrotic condition in which DNA damage causes cell cycle arrest via TP53 (p53) [93]. A similar mechanism may explain our results as DNA damage was enriched in our dataset. Interestingly expression of TP53 (p53) was strongly reduced in glaucomatous TFs which agrees with a study of pulmonary fibroblasts in which expression of TP53 was reported to be reduced in IPF patients [94]. Cyclin expression is in part regulated by transcription regulators of the E2F family [95, 96] and E2F1, E2F3, E2F4 were all downregulated. Apart from regulating several Cyclins, members of the E2F family of transcription regulators are also known to regulate their own transcription resulting in a positive feedback loop so it seems these regulators are at a stable low state in glaucomatous TFs.

The unfolded protein response (UPR) is associated with both POAG and fibrosis and was also found to be altered in our results. In POAG, activation of the UPR has been demonstrated in trabecular meshwork tissue in which expression of HSPA5, GRP94, ATF-4, ERO-1α and CHOP were all increased [97] and can be induced in trabecular meshwork cells when treated with TGF-β1 [56]. Activation of the UPR is also strongly associated with several fibrotic conditions [98, 99]. Our results show upregulation of DDIT3 (CHOP) which is associated with prolonged ER stress [99]. Expression of DDIT3 (CHOP) can be regulated by all three branches of UPR namely via EIF2AK3 (PERK), ATF6 and ERN1 (IRE1α) [100]. In our case ATF6B and it’s associated protease MBTPS1S1P (S1P) were upregulated suggesting that ATF6 may have caused upregulation of DDIT3. Further upstream, oxidative stress can activate several MAPKs such as JNK leading to upregulated expression of DDIT3 and induction of the UPR. Interestingly, increased expression of DDIT3 (CHOP) was also reported *in vitro* in human lung epithial cells after treatment with BAC [92]. Overall, our results suggest that glaucomatous Tenon’s ocular fibroblasts have been subjected to prolonged ER stress and activation of the UPR which could have been caused by exposure to cytokines, growth factors or ROS associated with POAG pathology and may be further exacerbated by exposure to glaucoma drugs and glaucoma drugs preservatives.

Both the intrinsic and extrinsic apoptotic pathways were altered in glaucomatous TF cells when compared to normal cells. Apoptosis in fibrotic conditions has been reported to be altered in myofibroblasts with respect to fibroblasts in the sense that myofibroblasts are primed for apoptosis but manage to escape this fate, for instance by upregulating pro-survival proteins such as BCL2L1 (BCL-XL) [34]. Evasion of apoptosis by myofibroblasts has been addressed by a class of drugs called BH3 mimetics which promote myofibroblast apoptosis thus reducing fibrosis [101]. Changes in pro-survival gene expression were also observed in our results: BCL2L2 (also known as BCL-W) was upregulated which may point to a similar apoptosis-evading mechanism because BCL2L2 suppresses effector BAX. BCL2L1 (BCL-XL) on the other hand was unchanged in our results. Apoptotic resistance is also affected by extracellular signal molecules TGF-β1 (TGFB1) and EDN1 (endothelin, ET-1). As noted before, TGF-β1 is upregulated in our results while expression of EDN1 was unchanged. TGF-β1 can be both pro-apoptotic or anti-apoptotic depending on cell type and context. For myofibroblasts TGF-β1 has been reported to act as a pro-survival cytokine and can inhibit sensitiser BAD via the FAK-AKT pathway [102, 103]. TGF-β1 also influences the extrinsic apoptotic pathway by inhibiting FAS-FASL signalling [34]. Biomechanical signalling is yet another survival signal affecting the myofibroblast apoptotic pathway and relaxation of the ECM can trigger apoptosis in fibroblasts [104]. This type of signalling is mediated via integrins and especially integrin subunits β1 (ITGB1) and αν (ITGAV), both of which were upregulated in our results. Integrins can also mediate the force-dependent release of TGF-β1 from the ECM which may be another pro-survival mechanism [34]. A Tenon’s ocular fibroblast cultured *in vitro* in a regular tissue culture flask will likely not experience the same mechanical forces compared to its normal *in vivo* environment so it could be that apoptosis is affected by the *in vitro* culturing conditions in our experiment. Although there is a phenomenon called biomechanical memory lasting for several weeks [105] which may also play a role here. Apoptosis is highly associated with the pathogenesis of glaucoma, primarily via cell death of retinal ganglion cells but also in other ocular tissues such as the trabecular meshwork [106]. Several studies have reported changes in apoptosis in ocular tissues due to BAC treatment [10, 92, 107, 108] indicating that BAC treatment may also contribute to the observed changes in apoptotic genes in glaucomatous Tenon’s ocular fibroblasts when compared to normal fibroblasts. One study compared apoptosis in untreated versus treated POAG patients and demonstrated increased apoptosis in the trabecular meshwork and iris from treated patients suggesting that the treatment itself may increase apoptosis [109].

Overall glaucomatous TF cells show altered expression of genes associated with apoptosis which may be due to a combination of cytokines and ROS originating from POAG itself and topical glaucoma drug ingredients. Glaucomatous TF cells may resist apoptosis by both upregulation of pro-survival genes such as BCL2L2 and by means of autocrine TGF-β1 signalling similar to other myofibroblasts.

Chronic low-level inflammation plays a key role in glaucoma [110–112] and also in the pathogenesis of fibrotic disorders [113, 114]. Our results demonstrated changes in inflammatory state when comparing glaucomatous to normal TF cells. In keeping with our findings expression of the pro-inflammatory cytokine IL6 (IL-6) has been shown to be increased in retinal tissue, trabecular meshwork and aqueous humour of glaucoma patients when compared to normal controls [111] and expression of this cytokine is also increased in several fibrotic conditions [115, 116]. Furthermore, increased preoperative levels of IL6 in the aqueous humour are correlated with poor glaucoma surgery outcome [117]. IL6 upregulation may be explained by activation of the NF-κB pathway via toll-like receptors [118]. With respect to the TLR pathway our results showed upregulation of TLR4 which has also been shown to play a role in fibroblast activation [119] and in glaucoma [111, 120, 121]. Another important inflammatory pathway is the TNF pathway which can also lead to NF-κB activation [122]. Our results indicated upregulation of TNF-α receptor TNFRSF1A and furthermore it has been reported that the concentration of TNF-α in the aqueous humor of glaucoma patients is elevated [123] so it is conceivable that this pathway was activated in glaucomatous TF cells.

Overall, we observed increased fibroblast to myofibroblast transdifferentiation in glaucomatous TFs when compared to normal TFs. This transdifferentiation may have been induced by POAG-associated ROS, cytokines and growth factors such as TGF-β2 originating from the aqueous humour and may be further exacerbated by ingredients of topical glaucoma drugs such as BAC and polyquat.

It is not clear from the results if POAG pathology itself or topical glaucoma drug ingredients are the dominant factor causing transdifferentiation in glaucomatous TFs, most likely it is a combination of both. The resulting myofibroblast phenotype shows increased ECM synthesis and remodelling, altered autophagy, increased mitochondrial fission, changes in the antioxidant system, cell cycle arrest, inflammation, upregulated unfolded protein response and altered apoptotic pathways. Furthermore, the myofibroblast phenotype seems to remain in its fibrotic state via a positive feedback loop due to autocrine TGF-β1 and IL6 signalling in addition to upregulation of transcription factors such as ZEB1 and ZEB2.

Our results indicate that glaucomatous TFs are already in a fibrotic myofibroblast state before glaucoma filtration surgery which may compromise a favourable outcome of this procedure [15]. This may also add to the variability of the surgical outcome because the preoperative Tenon’s ocular fibroblast state may likely range from fairly normal to fibrotic. This has implications for clinical practice in that surgical outcomes are likely to be impacted by the duration of the glaucomatous state, topical medication (number, duration and preserved or unpreserved) and epigenetic and genetic factors. Earlier surgical interventions with glaucoma filtration surgery or minimally invasive glaucoma surgery (MIGS) devices may result in improved outcomes but further research is required in this area [124–126]. Concomitant tissue assessment at the time of surgery may also predict the degree of fibrotic activation and epigenetic/genetic risks of fibrosis to direct more personalised therapeutic interventions [127].

Several of the identified pathways and associated genes in this work could be utilised to find suitable drug targets to reduce both preoperative and postoperative fibrosis and indeed several genes are already being investigated to improve the outcome of glaucoma filtration surgery such as the TGF-β pathway, VEGFs, PDGF, MMPs, SPARC and p53 [12, 128, 129]. Furthermore, it may also be attempted to reverse myofibroblast transdifferentiation prior to surgery e.g. by metformin treatment [130, 131]. In addition, given the fact that glaucomatous TFs are already in a profibrotic myofibroblastic state is clinically relevant to animal studies of glaucoma filtration surgery investigating novel therapeutic approaches. In these animal models the Tenon’s ocular fibroblasts have not been exposed to the profibrotic environment of the glaucomatous state. This may be one explanation why promising therapeutic candidates from animal studies fail in clinical trials in glaucoma patients [132]. The use of Tenon’s ocular fibroblast/bulbar conjunctiva interface biomimetic models seeded with GTFs may provide a better model system to investigate new anti-fibrotic therapies [133].

## Conclusions

To our knowledge this is the first study reporting a genome-wide transcriptome comparing glaucomatous to non-glaucomatous Tenon’s ocular fibroblasts. Our results indicate that glaucomatous Tenon’s ocular fibroblasts are transdifferentiated into myofibroblasts with associated changes in ECM remodelling, autophagy, mitochondrial morphology, antioxidant system, cell cycle, inflammation, unfolded protein response and apoptosis. These results may provide insight into wound-healing and fibrosis associated with glaucoma filtration surgery and support the development of novel therapeutic interventions to improve the outcome of glaucoma filtration surgery.

## Supporting information

S1 Fig Principal component analysis of RNA-Seq result. Two-dimensional Principal Component Analysis (PCA) plot showing grouping of normal samples C1..C6 in blue and glaucomatous samples C7..C12 in orange. All glaucomatous samples cluster together while the set of normal samples shows more spread, especially samples C3 and C5, but is still well separated from the glaucomatous set of samples.
(TIF)

S2 Fig Extracellular matrix gene-enrichment heatmap. Heatmap showing genes (FDR<0.05) associated with key enrichment terms related to remodelling of the extracellular matrix. Heatmap colours correspond to fold change expressed as log2FC which has been limited to a range of [–4,4] for clarity.
(TIF)

S3 Fig Autophagy gene-enrichment heatmap. Heatmap showing genes (FDR<0.05) associated with key enrichment terms related to autophagy. Heatmap colours correspond to fold change ex-pressed as log2FC which has been limited to a range of [–4,4] for clarity.
(TIF)

S4 Fig Unfolded protein response gene-enrichment heatmap. Heatmap showing genes (FDR<0.05) associated with key enrichment terms related to the unfolded protein response. Heatmap colours correspond to fold change ex-pressed as log2FC which has been limited to a range of [–4,4] for clarity.
(TIF)

S1 Table Differential expression of genes. This table contains fold-change (log2FC), false discovery rate (FDR), p-value and counts per million mapped reads (CPM) for all genes expressed above a threshold of 100 reads averaged over samples.
(XLSX)

S2 Table Enrichment results. This table contains all enrichment results using a list of differentially expressed genes (FDR<0.05) as input for databases WikiPathways, KEGG, Reactome, MSigDB Hallmark genesets, Gene Ontology (GO) biological processes and molecular functions. Each entry contains a metric of significance, a genelist relevant for the enrichment result and a cluster to which the enrichment term has been assigned, if applicable.
(XLSX)

S3 Table Donor information. Table containing donor information for the 12 Tenon’s capsule tissue samples.
(DOCX)
