# Supplementary figures and images for: Genome-wide RNA sequencing of ocular fibroblasts from glaucomatous and normal eyes: Implications for glaucoma management
Source: PLoS One. 2024 Jul 11;19(7):e0307227. doi: 10.1371/journal.pone.0307227 (PMC11239048; doi:10.1371/journal.pone.0307227)

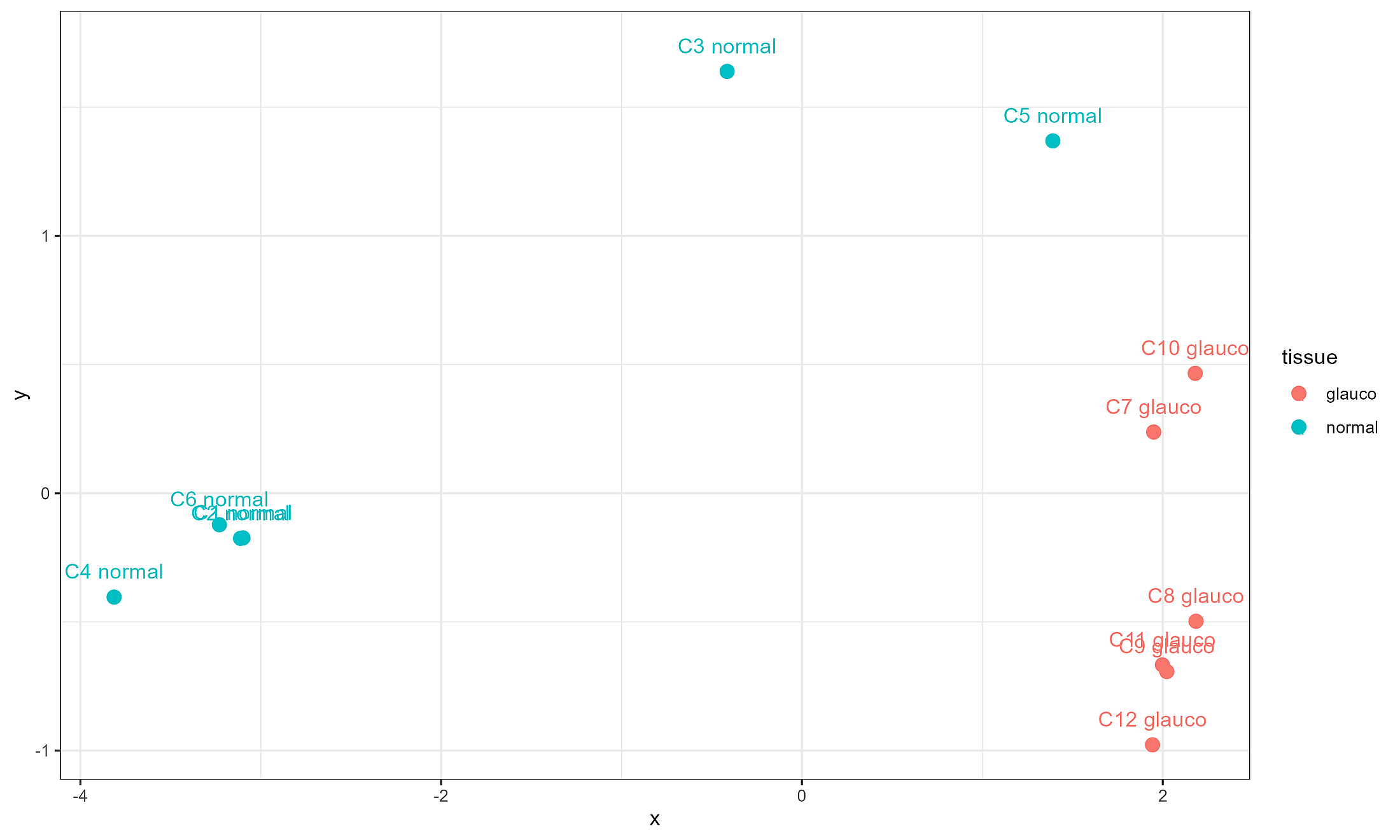

Supplement: S1 Fig — Two-dimensional Principal Component Analysis (PCA) plot showing grouping of normal samples C1..C6 in blue and glaucomatous samples C7..C12 in orange. All glaucomatous samples cluster together while the set of normal samples shows more spread, especially samples C3 and C5, but is still well separated from the glaucomatous set of samples. (TIF) [file pone.0307227.s001.tif]

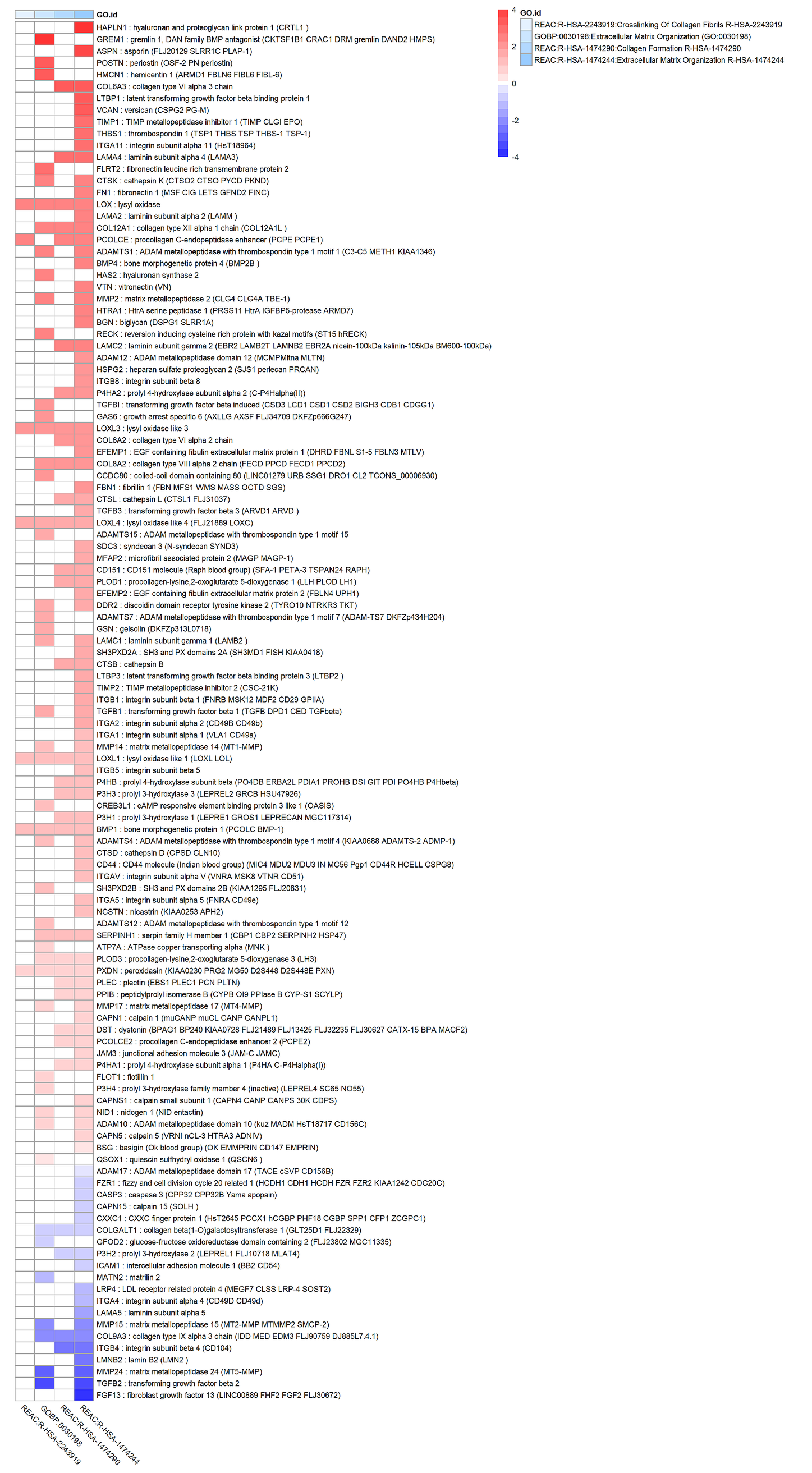

Supplement: S2 Fig — Heatmap showing genes (FDR<0.05) associated with key enrichment terms related to remodelling of the extracellular matrix. Heatmap colours correspond to fold change expressed as log2FC which has been limited to a range of [–4,4] for clarity. (TIF) [file pone.0307227.s002.tif]

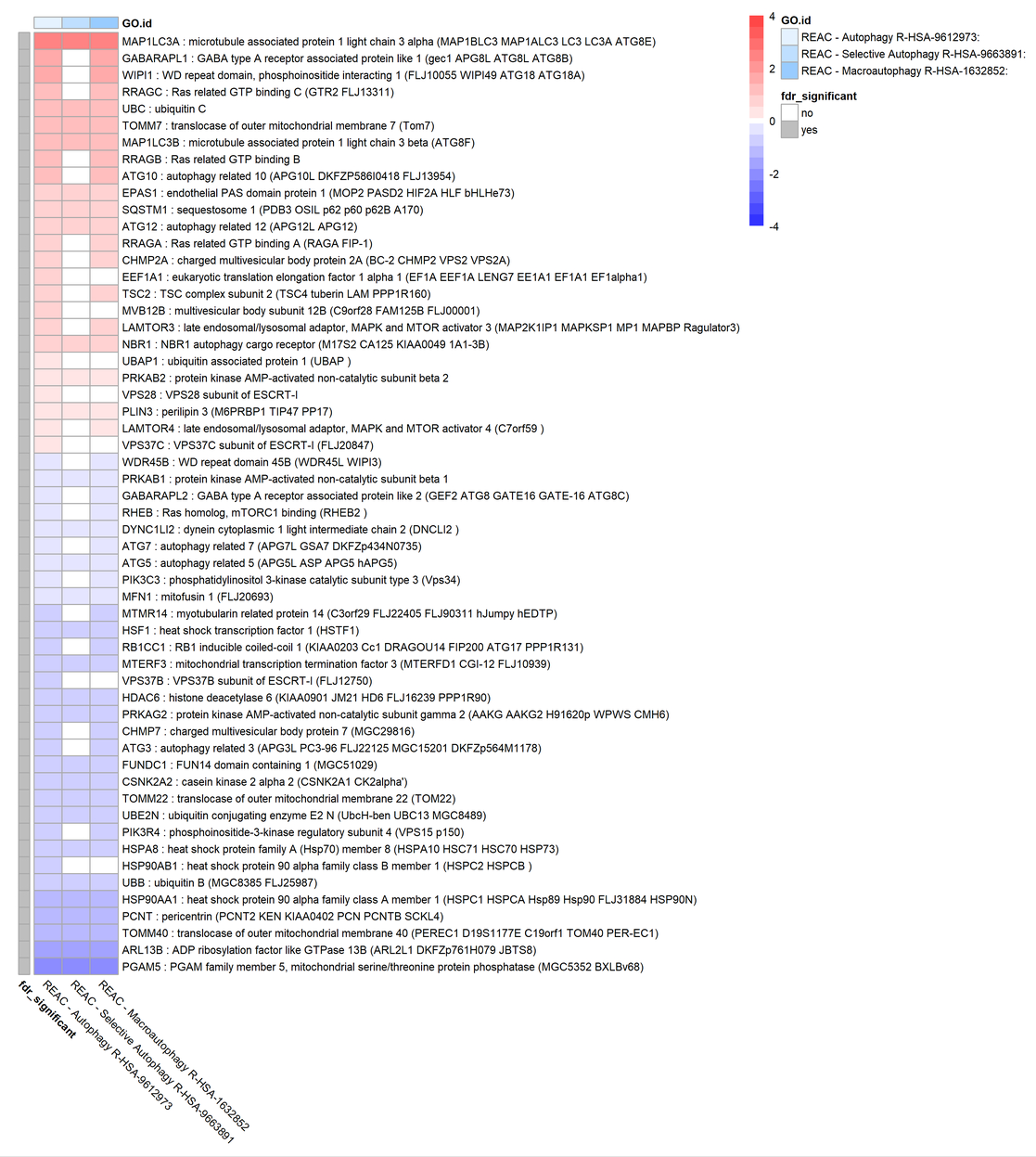

Supplement: S3 Fig — Heatmap showing genes (FDR<0.05) associated with key enrichment terms related to autophagy. Heatmap colours correspond to fold change ex-pressed as log2FC which has been limited to a range of [–4,4] for clarity. (TIF) [file pone.0307227.s003.tif]

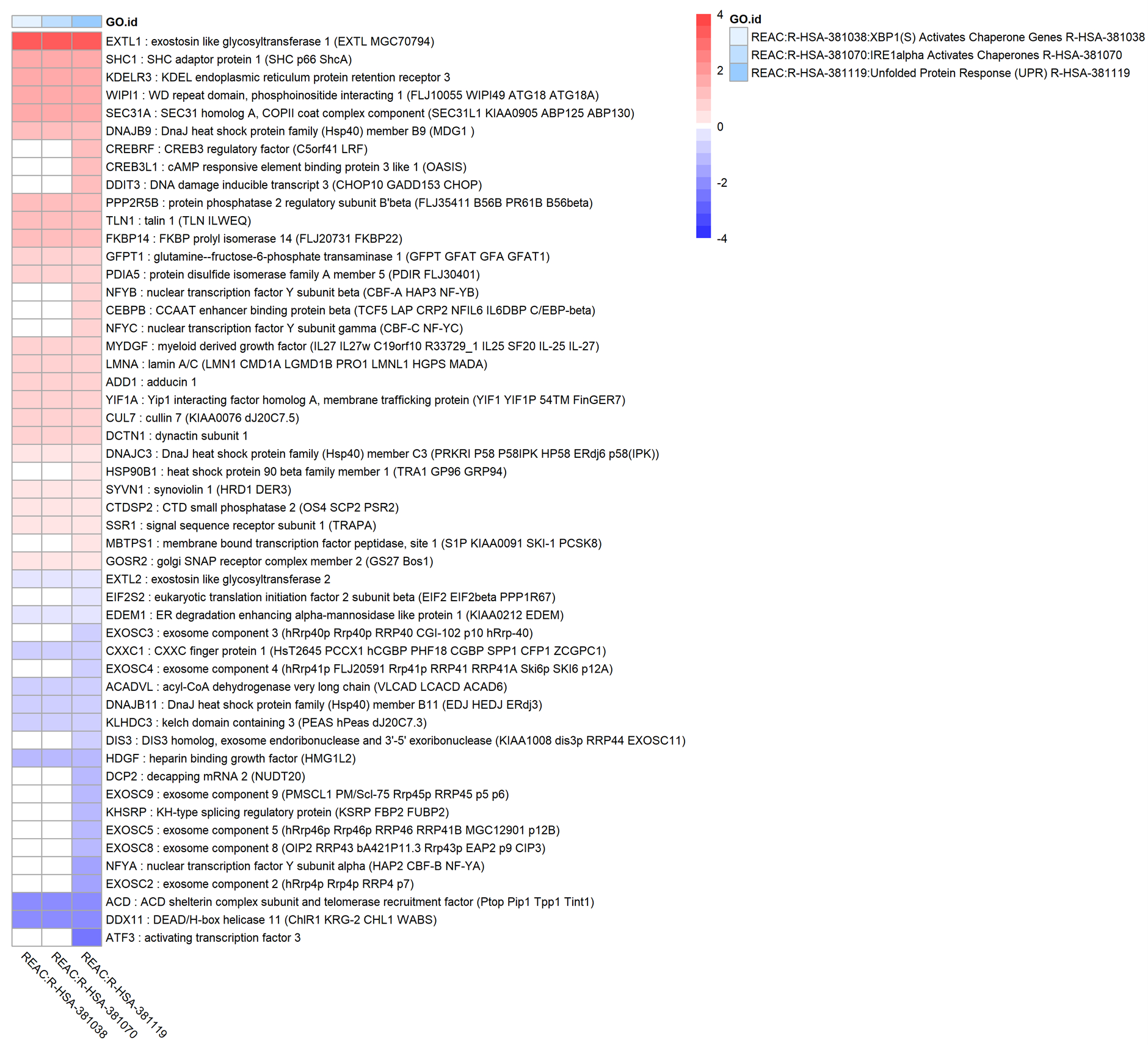

Supplement: S4 Fig — Heatmap showing genes (FDR<0.05) associated with key enrichment terms related to the unfolded protein response. Heatmap colours correspond to fold change ex-pressed as log2FC which has been limited to a range of [–4,4] for clarity. (TIF) [file pone.0307227.s004.tif]
