# Supplementary material for: Genome-wide RNA sequencing of ocular fibroblasts from glaucomatous and normal eyes: Implications for glaucoma management
Source: PLoS One. 2024 Jul 11;19(7):e0307227. doi: 10.1371/journal.pone.0307227 (PMC11239048; doi:10.1371/journal.pone.0307227)
Supplement: S3 Table — Table containing donor information for the 12 Tenon’s capsule tissue samples. (DOCX) [file pone.0307227.s007.docx]

# S3 Table – Donor information

| sample ID | sample name | ethnicity | sex | age at surgery |
| --- | --- | --- | --- | --- |
| C1_NTF | control | European descent | F | 75 |
| C2_NTF | control | European descent | F | 72 |
| C3_NTF | control | European descent | F | 54 |
| C4_NTF | control | European descent | F | 74 |
| C5_NTF | control | European descent | M | 67 |
| C6_NTF | control | European descent | F | 82 |
| C7_GTF | glaucomatous | European descent | M | 51 |
| C8_GTF | glaucomatous | European descent | M | 48 |
| C9_GTF | glaucomatous | European descent | F | 73 |
| C10_GTF | glaucomatous | European descent | M | 72 |
| C11_GTF | glaucomatous | European descent | F | 70 |
| C12_GTF | glaucomatous | European descent | M | 80 |
